# Phylogenomics of the *Hyalella* amphipod species-flock of the Andean Altiplano

**DOI:** 10.1038/s41598-020-79620-4

**Published:** 2021-01-11

**Authors:** Francesco Zapelloni, Joan Pons, José A. Jurado-Rivera, Damià Jaume, Carlos Juan

**Affiliations:** 1 Department of Biology, University of the Balearic Islands, Ctra. Valldemossa km 7’5, 07122 Palma de Mallorca, Balearic Islands Spain; 2 IMEDEA (CSIC-UIB), Mediterranean Institute for Advanced Studies, C/ Miquel Marquès 21, 07190 Esporles, Balearic Islands Spain

**Keywords:** Evolutionary genetics, Molecular evolution, Phylogenetics, Speciation, Taxonomy

## Abstract

Species diversification in ancient lakes has enabled essential insights into evolutionary theory as they embody an evolutionary microcosm compared to continental terrestrial habitats. We have studied the high-altitude amphipods of the Andes Altiplano using mitogenomic, nuclear ribosomal and single-copy nuclear gene sequences obtained from 36 *Hyalella* genomic libraries, focusing on species of the Lake Titicaca and other water bodies of the Altiplano northern plateau. Results show that early Miocene South American lineages have recently (late Pliocene or early Pleistocene) diversified in the Andes with a striking morphological convergence among lineages. This pattern is consistent with the ecological opportunities (access to unoccupied resources, initial relaxed selection on ecologically-significant traits and low competition) offered by the lacustrine habitats established after the Andean uplift.

## Introduction

Lakes with an uninterrupted history of more than 100,000 years (ancient lakes) may be considered as natural laboratories for evolutionary research as they constitute hotspots of aquatic animal speciation and phenotypic diversity^1^. Changes in lake size and episodes of desiccation are considered to be critical factors in the speciation and extinction of lake faunas, with the creation of new habitats after lake expansions as the primary driver of intra-lake diversification^2–4^. For instance, cichlid radiations in the East African Lakes seem to have been triggered by lake expansions after periods of intense desiccation, with the surviving species filling up empty niches after lake refilling^2^.

**Figure 1:**
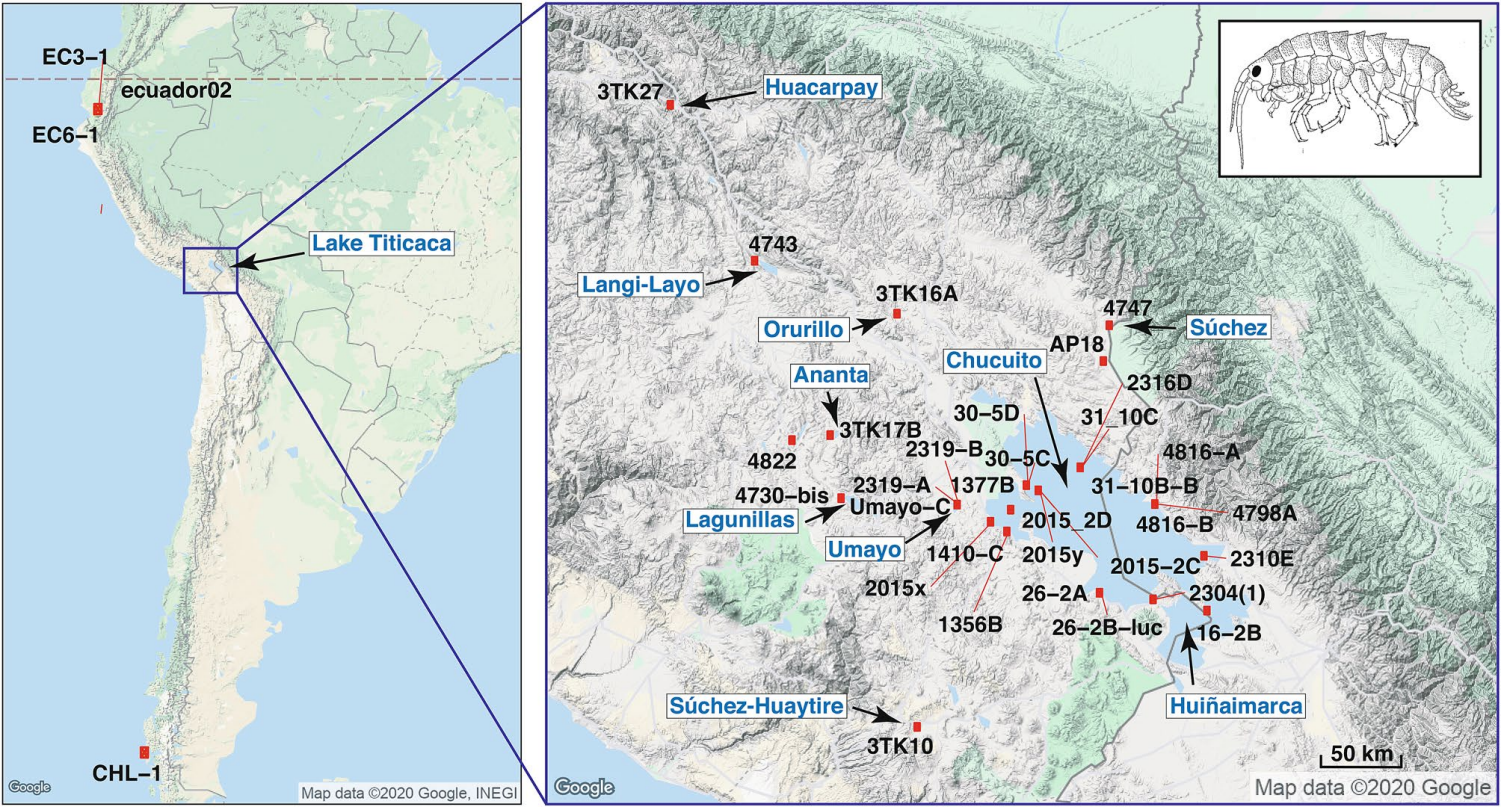
Map of South America showing the location of the Andean Altiplano, the *Hyalella* sampling sites and the names of main water bodies mentioned in the text. Codes identify samples used in the analyses. See Table 1 for additional information on samples. Inset shows a drawing of *Hyalella solida* by one of the co-authors (D. Jaume). Map was drawn using the package ‘ggmap’ version 3.0.0.902 (https://cran.r-project.org/web/packages/ggmap/) in R version 3.6.3 (https://www.R-project.org/). The maps of South America and the Andean Altiplano were obtained with Google Maps (Map data 2020 Google) using the function ‘get_map’ in the ‘ggmap’ package.

Lake Titicaca, located in the Andean high plateau in the central Andes of Perú, Bolivia and Argentina at an elevation of 3806 m, is part of an extensive intermontane endorheic area of about 200,000 km^2^ that also includes Lake Poopó, and the salt flats of Coipasa and Uyuni^5^ (Fig. 1). The Titicaca is the only ancient Lake present in South America, with a presumed age of between 3 and 2 million years (Ma)^5,6^ ensuing the end of the uplift of the northern Andean Altiplano (at 5.4 ± 1.0 Ma^7^). The Lake consists of two main sub-basins, the northern one (Lake Chucuito) attains a maximum depth of 285 m, being separated from the southern sub-basin (Lake Huiñaimarca) by the Tiquina Strait (Fig. 1). Like many other ancient lakes such as the East African ones, the Altiplano lakes have experienced a complex palaeo-environmental history as the water level was subjected to at least three significant expansions from the Early to Middle Pleistocene. These shifts resulted in the joining of the different lake basins of the Altiplano into a single hydrological unit^8–10^. Besides, during global interglacial periods, the water level dropped considerably, resulting in an increase in water salinity, and a closed-basin configuration of the Titicaca^11^. These significant lake-level fluctuations split apart the Titicaca basin into three palaeolakes in recent times (8000 year before present), presumably influencing the population dynamics of the lacustrine taxa found therein^12^.

Several aquatic species-flocks (e.g. gastropod molluscs, crustacean amphipods, cyprinodontid fish) dwell in the Titicaca and its peripherical water bodies^4,6,13–15^. The genus *Hyalella* S. I. Smith, 1874 (Fam. Hyalellidae Bulycheva, 1957) constitutes the only epigean amphipod lineage present in continental waters of South America^16^, with at least 18 species recorded in the Andean Altiplano, including Lake Titicaca^17–20^. Previous studies using mitochondrial cytochrome c oxidase subunit I (*cox1*) DNA sequences supplemented with a nuclear marker demonstrated that the *Hyalella* in the Titicaca are polyphyletic, and divide into five major genetic lineages embedded in the broader South American *Hyalella* phylogeny^14,15^. Several independent colonizations deriving from South American lineages that diverged from each other in the last 15–20 My seem to have populated the Andean Altiplano, with evidence of the occurrence of intra-lacustrine diversification detected in at least two of the clades^14,15^. In a previous work, we applied molecular species delimitation criteria concluding that at least one-third of the *Hyalella* species diagnosed in that study are likely endemic to the Titicaca and neighbouring water bodies^15^. We also uncovered a disagreement between morphology and genetic data in the Titicacan *Hyalella*, with cases of same morpho-species displaying very distinct mitochondrial haplotypes, while others diagnosed as belonging to the same *cox1* molecular operational taxonomic unit (MOTU). No significant discordances between mitochondrial and nuclear data were observed in Titicacan amphipods by either the Adamowicz et al.^14^ or Jurado-Rivera et al.^15^ surveys. Morphological conflict with molecular phylogenies has also been detected in amphipods of ancient Lake Baikal^21^. These disagreements were proposed to derive by rapid morphological and ecological differentiation coupled with phenotypic convergence^21–23^.

The use of mitochondrial genomes for phylogenetics in metazoans, including crustaceans, has exploded in recent years due to the advances experienced in genomic techniques^24^. Although the usefulness of mitochondrial sequences as a sole genetic marker has been questioned^25^, they are still widely used in phylogenetic inference, species delimitation or identification and, in conjunction with nuclear multilocus data, to detect mito-nuclear discrepancies derived from hybridization/introgression events^26^. In the present study, we contribute the mitogenomes and the SSU, plus LSU nuclear ribosomal DNA sequences of 32 *Hyalella* High Andes amphipods. Also, we identify single-copy nuclear orthologous genes present as a single copy in most species (orthogroups) to investigate their evolutionary origin and phylogenetic relationships.

Our main goal is to establish an evolutionary scenario for amphipods of the Andean plateau by achieving the following objectives:

1. to infer a robust phylogeny of *Hyalella* in the Altiplano, sampling their full geographic distribution in the two primary Titicaca sub-basins, main satellite lakes, and other high-altitude water bodies in the area;
2. to estimate tree node ages and date of colonization of the Lake by the different lineages, testing the hypothesis of occurrence of recent intra-lacustrine radiation in particular clades; and
3. to test the occurrence of mito-nuclear discordances and the congruence of sequence data with morphological characters under a phylogenetic framework.

The results obtained should shed light on questions such as (1) did clade diversification occur synchronously among different lineages? (2) are diversifications concordant with the time-frame of endorheic basins establishment ensuing the northern Andean Altiplano uplift? Furthermore, (3) do the emergence of morphological key-innovation traits explain the Andean amphipod radiation or were the ecological opportunities provided by the emergence of island-like mountain lacustrine habitats the main factor implied in their diversification?

## Results

### Mitogenome phylogeny

**Figure 2:**
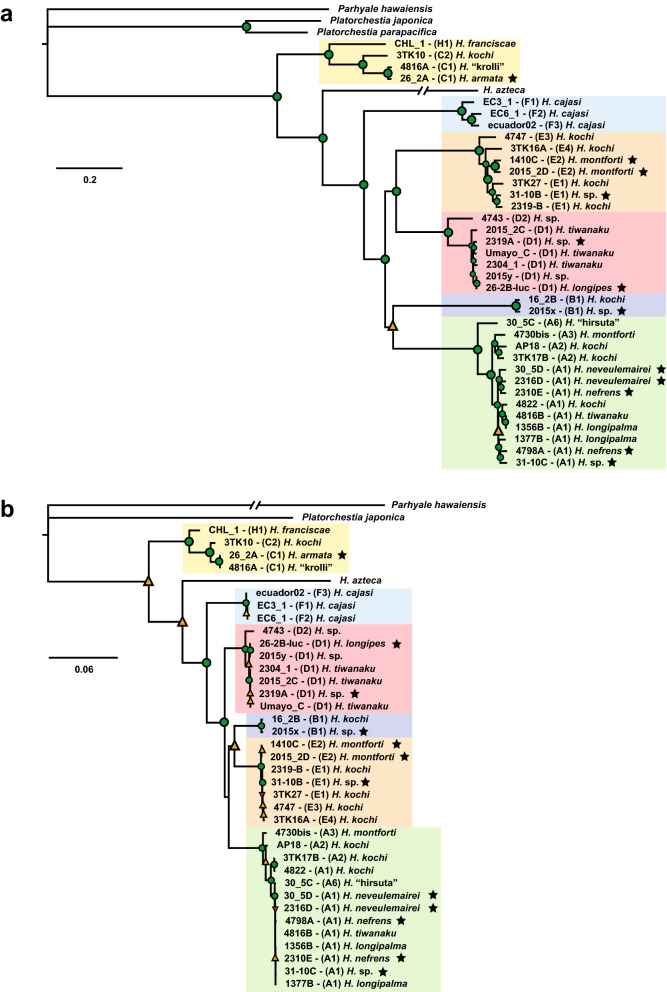
(**a**) Maximum Likelihood phylogeny based on the 13 mitochondrial protein-coding genes of *Hyalella* and outgroup taxa. (**b**) Maximum Likelihood phylogeny based on LSU and SSU nuclear rDNA sequences. Colours for different lineages define the main clades (see main text for details and Fig. 4). Trees are available in nexus format in the GitHub repository (https://github.com/Frazapel/Hyalella-amphipod-Phylogenomics). Nodes in a and b are marked with different colours to indicate bootstrap support: green circles (maximum nodal support, i.e. 100), orange triangles < 96 and red triangles < 80%. Stars denote species with armoured spiny morphologies.

Thirty-five new *Hyalella* complete or partial mitogenomes representing species from each major clade in the Titicaca Altiplano area plus samples from Ecuador and Chile were obtained (Table 1). Details of mitogenome size, sequence completeness, gene order and A + T content are given in Supplementary Text 1 and Supplementary Table 1). The alignment of the 13 mitochondrial protein-coding genes (PCGs) of the South American *Hyalella* species plus the North American *H. azteca* and three outgroup taxa (*Parhyale hawaiensis, Platorchestia japonica* and *Platorchestia parapacifica*) comprised 11,073 bp (available at https://github.com/Frazapel/Hyalella-amphipod-Phylogenomics). Xia’s test showed low levels of substitution saturation except for third coding positions in which moderate levels of saturation were detected (Supplementary Text 1). The Maximum Likelihood (ML) phylogenetic tree robustly supports *Hyalella* as a monophyletic clade divided into two major lineages: one comprising *H. franciscae* (from southern Chile), and all the representatives of clade C, which consists of one not yet formally described species of the Titicaca with smooth body integument—*H.* “krolli”—that is sister to the heavily armoured Titicacan *H. armata*, and another sister lineage sampled at the western border of the Altiplano (Laguna Súchez-Huaytire) represented by a species with smooth body integument. The second highly supported major lineage encompasses the North American species *H. azteca,* and all the remaining South American species (Titicaca + Altiplano clades A, B, D and E plus the taxa from Ecuador clade F), with all subclades supported with maximum bootstrap values except the node relating clades A and B (that received a 95 bootstrap support value; Fig. 2a). None of the alternative topologies obtained under ML could be rejected by the Approximately Unbiased Test of Phylogenetic Tree Selection (AU test) (Supplementary Table 2) except for the one showing *H. azteca* as sister to the rest of South American *Hyalella*, although with a marginal *P* value (*P* = 0.0294). However, this hypothesis could not be rejected when the analysis did not include outgroups (*P* = 0.6230). The trees were also explored under a Bayesian framework at both nucleotide and amino acid levels implementing various nucleotide and codon substitution models (Supplementary Fig. 2a–j). The results of these analyses converged to the same or similar mitogenome tree topologies with minor differences in support values. Phylogenies based on single mitochondrial genes were less resolved and supported than those based on the concatenated genes, with the more informative *atp6, cob, cox1, cox3 nad1, nad3* and *nad4* genes rendering the same topology as the all genes dataset. In contrast, *cox2* and *nad5* supported an alternative topology in which *H. azteca* and the South American *Hyalella* are reciprocally monophyletic (Supplementary Fig. 2).

**Table 1:** Taxa included in the analysis.

Species	MOTU	Code	Locality	Coordinates	Altitude (m)	Depth (m)	Morphology
*H. nefrens*	A1	4798A	Titicaca, Escoma, Bolivia	− 15.73621 − 69.08813	3825	0–1	Armoured
*H. nefrens*	A1	2310E	Titicaca, Punta Khauani, Bolivia	− 16.01000 − 68.81900	3815	2	Armoured
*H. neveulemairei*	A1	30-5D	Titicaca, Isla Ticonata, Perú	− 15.63700 − 69.79200	3815	26	Armoured
*H. neveulemairei*	A1	2316D	Titicaca, Isla Soto, Perú	− 15.54300 − 69.49700	3815	12	Armoured
*H.* sp.	A1	31-10C	Titicaca, Isla Soto, Perú	− 15.54300 − 69.49700	3815	23	Armoured
*H. montforti*	A3	4730-bis	Altiplano: Laguna Lagunillas, Perú	− 15.70587 − 70.80633	4177	0–1	Smooth
*H. kochi*	A4	AP18	Altiplano: Río Súchez, Perú	− 14.98298 − 69.37053	4340	< 0.5	Smooth
*H. kochi*	A4	3TK17B	Altiplano: Laguna Ananta, Perú	− 15.37245 − 70.86678	4848	< 1	Smooth
*H. longipalma*	A5	1377B	Titicaca, Bahía de Puno, Perú	− 15.76547 − 69.87792	3815	9	Smooth
*H. longipalma*	A6	1356B	Titicaca, Salmonid culture station, Perú	− 15.88200 − 69.89800	3815	2.7	Smooth
*H. tiwanaku*	A7	4816-B	Titicaca, Escoma, Bolivia	− 15.73621 − 69.08813	3834	0–1	Smooth
*H. kochi*	A7	4822	Altiplano: near Laguna Pañe, Perú	− 15.40025 − 71.07559	4612	< 0.7	Smooth
*H.* “hirsuta”	A8	30-5C	Titicaca, Isla Ticonata, Perú	− 15.63700 − 69.79200	3815	26	Smooth
*H. kochi*	B1	16-2B	Titicaca, Isla Chipi, Bolivia	− 16.29700 − 68.80300	3820	1	Smooth
*H.* sp.	B1	2015x	Titicaca, Puno, Perú	− 15.82940 − 69.98800	3815	< 1	Armoured
*H. armata*	C1	26-2A	Titicaca, Chocasuyu, Perú	− 16.20300 − 69.39100	3815	23	Armoured
*H.* “krolli”	C1	4816-A	Titicaca, Escoma, Bolivia	− 15.73621 − 69.08813	3826	0–1	Smooth
*H. kochi*	C2	3TK10	Altiplano: Laguna Súchez-Huaytire, Perú	− 16.90758 − 70.38936	4612	< 1	Smooth
*H. tiwanaku*	D1	2304(1)	Titicaca, Yunguyo, Perú	− 16.23800 − 69.09800	3815	2	Smooth
*H. tiwanaku*	D1	2015-2C	Titicaca, Isla Amantani, Perú	− 15.66500 − 69.72660	3825	< 1	Smooth
*H.* sp.	D1	2319-A	Altiplano: Lago Umayo, Perú	− 15.73900 − 70.17100	3838	0–3	Armoured
*H. tiwanaku*	D1	Umayo-C	Altiplano: Lago Umayo, Perú	− 15.73900 − 70.17100	3870	0–3	Smooth
*H.* sp.	D1	2015y	Titicaca, Isla Amantani, Perú	− 15.66500 − 69.72660	3820	< 1	Smooth
*H. longipes*	D1	26-2B-luc	Titicaca, Chocasuyu, Perú	− 16.20300 − 69.39100	3819	23	Armoured
*H.* sp.	D2	4743	Altiplano: Lago Langui Layo, Perú	− 14.45231 − 71.27954	4316	0–2	Smooth
*H.* sp.	E1	31-10B-B	Titicaca, Isla Soto, Perú	− 15.54300 − 69.49700	3820	23	Armoured
*H. kochi*	E1	2319-B	Altiplano: Lago Umayo, Perú	− 15.73900 − 70.17100	3855	0–3	Smooth
*H. kochi*	E1	3TK27	Lago Huacarpay (Cuzco), Perú	− 13.62381 − 71.74054	3124	< 1	Smooth
*H. montforti*	E2	1410-C	Titicaca, Salmonid culture station, Perú	− 15.88200 − 69.89800	3820	5	Armoured
*H. montforti*	E2	2015_2D	Titicaca, Isla Amantani, Perú	− 15.66500 − 69.72660	3820	< 1	Armoured
*H. kochi*	E3	4747	Altiplano: Laguna Súchez, Perú	− 14.79379 − 69.33891	4646	0–1	Smooth
*H. kochi*	E4	3TK16A	Altiplano: Laguna Orurillo, Perú	− 14.73241 − 70.49987	3893	< 1	Smooth
*H. cajasi*	EC1	EC3-1	Laguna Azul, Cuenca, Ecuador	− 2.78849 − 79.24602	4034	< 1	Smooth
*H. cajasi*	EC2	EC6-1	near Laguna Luspa, Cuenca, Ecuador	− 2.79819 − 79.26298	3855	< 1	Smooth
*H. cajasi*	EC3	ecuador02	Laguna Cardenillo, Cuenca, Ecuador	− 2.78188 − 79.24730	4170	< 1	Smooth
*H. franciscae*	CH1	CHL-1	Isla Madre de Dios, Chile	− 50.10300 − 75.24400	256	< 1	Smooth

Details on their putative taxonomic placement, mitochondrial Molecular Operational Taxonomic Units (Jurado-Rivera et al.^15^) and geographic origin are indicated.

### Nuclear rDNA phylogeny

The alignment of the small and large nuclear ribosomal DNA sequences were 2395 and 5437 bp in length, respectively, after removal of long species-specific insertions present in the outgroups and *H. azteca* (available at https://github.com/Frazapel/Hyalella-amphipod-Phylogenomics). The ML trees obtained showed a backbone similar to the mitochondrial phylogeny, either based on MAFFT alignments or from those considering secondary RNA structures or Bayesian implementing the doublet model. However, some differences were affecting terminal tips and lower support was obtained at some deep nodes (Fig. 2b). In particular, the nuclear rDNA phylogeny provides relatively low support for the relationship between *H. azteca* and the South American clades excluding clade C (bootstrap value = 93), with low confidence for the node relating the two main *Hyalella* lineages (clade C and the remaining *Hyalella* lineages) (bootstrap value = 81) (Fig. 2b). The removal of poorly aligned positions and divergent regions and recoding gaps as binary characters irrespective of their length produced nearly the same phylogenetic relationships with slight variations on tips of the tree.

### Nuclear phylogeny based on single-copy nuclear genes

**Figure 3:**
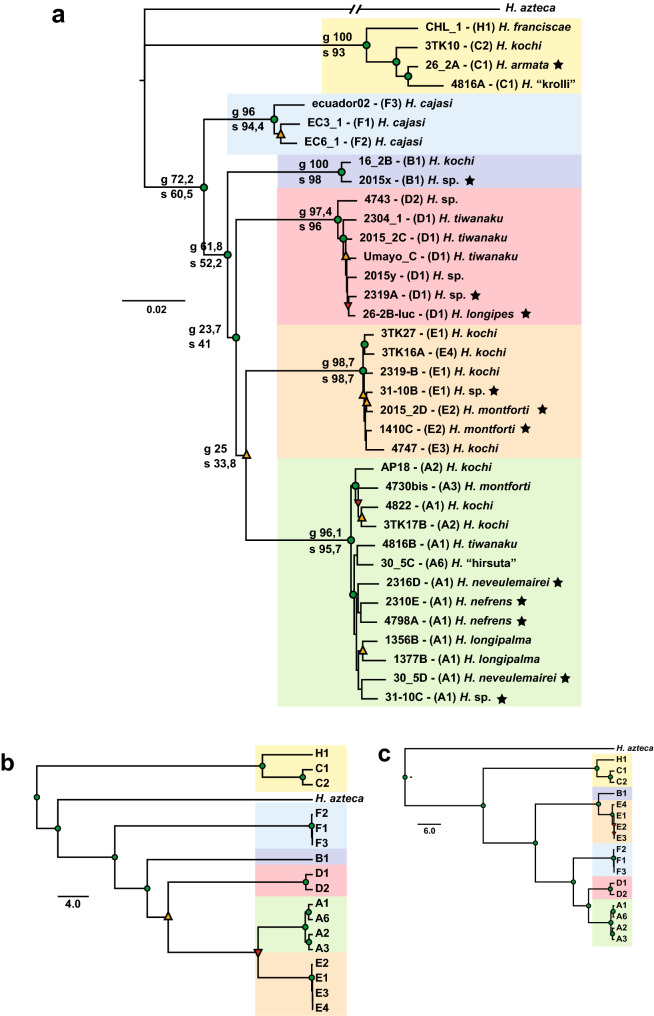
(**a**) Nuclear phylogeny based on the single-copy nuclear genes supermatrix. Node numbers above and below main branches indicate gCF and sFC support values respectively as the proportion of genes (gCF) and sites (sFC) that are concordant with any particular branch in the supermatrix. Code color for ML bootstrap support and stars as in Fig. 2. (**b**) Species tree based on multispecies coalescent model (MSC) using data of the 76 single-copy nuclear gene-fragments. (**c**) MSC tree based on single-copy nuclear gene-fragments plus the 13 mitochondrial protein-coding genes as an extra “gene”. For both (**b**,**c**) the terminals were classified into species following results by Jurado-Rivera et al.^15^, letters and numbers on tips refer to Molecular Taxonomic Operational Units. Trees are available in nexus format in the GitHub repository (https://github.com/Frazapel/Hyalella-amphipod-Phylogenomics). Code colour for nodal Bayesian posterior probability values in b) and c) as in Fig. 2.

A total of 76 single-copy nuclear gene segments present in the 36 low-coverage *Hyalella* genomic libraries were retrieved using Orthofinder^27^. BLAST similarity searches showed that 53 orthologous gene fragments matched known or predicted proteins of *H. azteca* (Supplementary Text 1 and Supplementary Table 3). The ML phylogeny obtained from the concatenated supermatrix (34,557 bp with a mean of 10.7% missing data, mainly due to missing orthologous in *H. azteca*) displayed the same six highly supported clades as the mitochondrial and nuclear ribosomal analyses, with clade C and *H. azteca* being part of a polytomy (Fig. 3a). The comparison of the nuclear protein-coding and mitogenome tree-topologies showed that they are roughly concordant (Baker’s index = 0.915; cophenetic index = 0.987), except for disagreement in the respective relationship of clades A, D and E (Supplementary Fig. 3). The Shimodaira-Hasegawa test (SH test) showed that a high number of gene trees were significantly preferred over the species tree (*P* < 0.05), i.e. they conflict with the concatenated tree-topology (Supplementary Table 4). This fact was further confirmed by gene and site-concordance factors^28^ except for the nodes defining main clades, where bootstrap and concordance factors were in complete agreement (Fig. 3a). The relative position in the base of the tree of Ecuador (F) and Altiplano (B) clades have maximum bootstrap support but moderate gene and site-concordance factors, suggesting that for these nodes there is a real tree discordant signal in the single locus trees. All other nodes tend to have different gene (gCF) and site-concordance factors (sCF) with often low values, implying this is due to a limited phylogenetic signal with short tree branches in most gene trees, in particular on tip nodes. The species tree based on multispecies coalescent models (MSC) was similar to the concatenated ML tree (Fig. 3b). The multispecies coalescent analyses that included the mitochondrial PCGs as another linkage group added to the orthologous nuclear sequences produced an alternative tree topology with maximum posterior probabilities at all relevant nodes (Fig. 3c). This topology supports the reciprocal monophyly of North and South American *Hyalella* and within the latter, distinguishes between two well-defined species-groups: clade C as sister to the clade embracing A + B + D + E + F. Finally, the species tree obtained with ASTRAL differed from the former phylogenetic hypotheses at various relevant nodes (Supplementary Fig. 4).

### Estimation of divergence times

**Figure 4:**
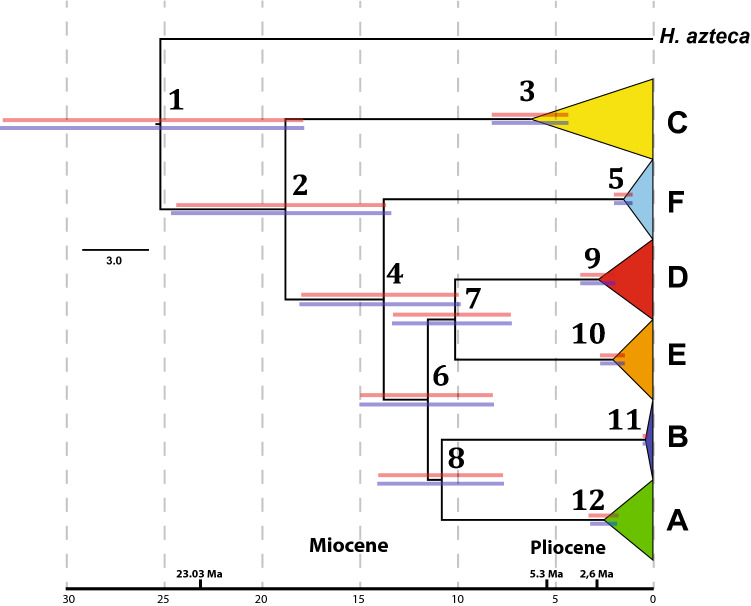
Simplified dated phylogeny (chronogram) of *Hyalella* based on the 13 mitochondrial protein-coding genes. The ages of two nodes were implemented separately as constraints to calibrate the relaxed molecular clock (MRCA of node A and tree root after Adamowicz et al.^14^). Blue and red bars above nodes indicate 95% higher posterior densities for node ages when using 2.8 ± 0.4 Ma as age constrain for clade A and 20.63 Ma for the tree root, respectively. Nodes are numbered for discussion in the main text (nodes 1–12). Trees are available in nexus format in the GitHub repository (https://github.com/Frazapel/Hyalella-amphipod-Phylogenomics).

Molecular clock analyses using mitogenomic data and Bayes Factors favoured the reciprocal monophyly of North and South American *Hyalella* as shown in the robust MSC tree based on mitogenomes and single nuclear genes. The two independent calibration schemes applied rendered similar age estimates along the phylogeny, showing congruence with each other as deduced from the result of cross-validation analysis (Fig. 4 and Table 2). The mean time for the first divergence within the South American taxa was estimated at ca. 19 Ma (node 2 in Fig. 4; 95% HPD interval 13–25 Ma). It represents the divergence time of the common ancestor of clade C and the remaining clades. The average time of the initial divergence within the Most Recent Common Ancestor (MRCA) of A, B, E and D lineages was estimated at 11.7 Ma (95% HPD interval 8–15 Ma using calibration 1) (node 6 in Fig. 4 and Table 2). For clades A, D and E the mean time of the first divergence were estimated to be almost synchronous at a narrow interval of 2.1–2.8 Ma (Nodes 9, 10 and 12) with 95% HPD intervals largely overlapping. The age for clade B resulted much younger (estimated age 0.4 Ma; node 11 in Fig. 4 and Table 2). These results suggest that species diversification of the different lineages in the Altiplano started mainly at the end of the Pliocene. The age of clade C is older (6.2–6.3 Ma; 95% HPD interval 4.4–8.4 Ma using calibration 1), although the age of Altiplano species-group in this clade is concordant with that obtained in the other lineages (2.6 Ma; 95% HPD 1.8–3.5, not shown).

**Table 2:** Estimated ages and 95% higher posterior densities (HPD) for each node in the best mitochondrial tree according to Bayes Factor scores obtained in BEAST under the two alternative calibration schemes (MRCA of node A and tree root after Adamowicz et al.^14^; see text for details).

Node	Node A calibration		Root age calibration	
	Node age (Mya)	HPD 95% (Mya)	Node age (Mya)	HPD 95% (Mya)
1	25.6	[18.111–33.911]	25.0	[17.63–32.647]
2	19.1	[13.587–25.025]	18.7	[13.518–20.009]
3	6.3	[4.389–8.367]	6.2	[4.338–8.164]
4	14.0	[9.985–18.357]	13.7	[9.801–17.679]
5	1.5	[1.062–2.019]	1.5	[1.058–1.99]
6	11.7	[8.257–15.246]	11.4	[8.3–14.913]
7	10.3	[7.327–13.548]	10.0	[7.128–13.017]
8	11.0	[7.737–14.316]	10.7	[7.639–13.87]
9	2.8	[1.956–3.77]	2.7	[1.914–3.662]
10	2.1	[1.468–2.736]	2.0	[1.454–2.688]
11	0.4	[0.258–0.529]	0.4	[0.254–0.521]
12	2.5	[1.861–3.26]	2.5	[1.747–3.247

## Discussion

### Phylogenomics of the Andean Altiplano *Hyalella*

Nuclear and mitochondrial phylogenomic analyses strongly support the hypothesis that the Titicaca species-flock originated from multiple colonization events from independent *Hyalella* ancestral lineages, with clades not showing marked relationships to particular sub-basins or regions in the main Lake or adjacent water bodies. Previous results using a short mitochondrial gene fragment (*cox1*) on a broad South American sampling of the genus pointed to the occurrence of at least five major distant monophyletic clades in the Titicaca and other waterbodies in the northern Altiplano^14,15^. Clades A, B, C and E were shown to be part of lineages displaying a wider South American distribution, while members of clade D were exclusive of the Altiplano^15^. The mitogenome phylogeny reported herein is fully compatible with the tree topology obtained based on the *cox1* fragment, showing that the South American *Hyalella* species sampled are divided into two distant ancestral lineages. A highly supported clade comprises clade C^14,15^, a lineage that includes the species examined from southern Chile (*H. franciscae*) plus two taxa from the northern Andean Altiplano (the heavily armoured Titicacan species *H. armata,* and another species with smooth body integument found at Laguna Súchez-Huaytire, on the western limit of the Altiplano). The other major lineage is composed of four northern Altiplano clades (clades A, B, D and E) plus a clade from the southern Ecuador highlands (clade F). The former clades have a wide distribution in the study area of Peru and Bolivia, as samples from each clade were collected in the two major basins of the Titicaca but also from peripheral lakes, lagoons and streams outside the main lake (Table 1; Fig. 2a and see Jurado-Rivera et al.^15^). The low sequence divergence, and the lack of resolution within clades A, B, D and E suggest the occurrence of rapid and recent diversification in the northern Altiplano.

The phylogenetic relationship of the South American clades with the North American *H. azteca* is ambiguous in the mitogenomic trees as the AU test could not confidently reject two competing tree topologies: (1) North and South American *Hyalella* as reciprocally monophyletic (as in Adamowicz et al.^14^ versus 2) clade C sister to the remaining clades (including *H. azteca*). This ambiguity may be caused by the long tree-branch leading to *H. azteca,* compared to the shorter branches displayed by the South American clades, and by a highly unbalanced representation of the North and South American *Hyalella* species in the phylogeny, as only the mitogenome of *H. azteca* is currently available for the North American lineages of the genus. The SSU and LSU ribosomal phylogenetic hypotheses were mostly consistent with the mitogenome-based tree concerning the main clades. However, less support was obtained for the interclade relationships (Fig. 2b). In particular, a weak support for the relationship of clade B to other clades. Nevertheless, the mitogenome and nuclear ribosomal trees agreed in the monophyly of the node from which all clades derive with the exclusion of the distant clade C. Trees obtained using single-copy nuclear genes, either from concatenated data or applying the multispecies coalescent model, confirmed previous results. However, interclade relationships were still sensitive to the method used. A comparison of the mitochondrial and nuclear phylogenies showed that discrepancies were also focalized at the tip nodes in clades A, E and D, likely reflecting shallow divergences and low phylogenetic signal at this level, in particular for single-copy highly conserved nuclear genes (Supplementary Fig. 3). The more robustly supported phylogeny obtained resulted from applying the multispecies coalescent model to the dataset including the mitochondrial protein-coding genes as a single linkage group plus the single-copy nuclear gene sequences (a total of 45.6 Kb of DNA sequence information). This topology supports North American (*H. azteca*), and South American *Hyalella* as reciprocally monophyletic, with clade C as sister to all other clades (Fig. 3c).

### Divergence times and palaeohydrology

Calibration of the molecular clock by two independent methods provided similar estimates for the split of the North and South American *Hyalella* at around 25 Ma, with Andean Altiplano lineages (node 2) inferred to date back to the early Miocene (Fig. 4 and Table 2). The diversification of Altiplano species within clades is much more recent in comparison, showing in three lineages time frames consistent with the presumed formation of Titicaca (between 3 and 2 Ma^6,29^). Thus, clades A, D and E seem to have diversified recently and relatively rapidly by intra-lacustrine diversification. Their estimated ages are posterior to the final uplift of the northern Andes during the late Pliocene or early Pleistocene 2–4 Ma, being coeval to paleolake Mataro, ancestor of current Lake Titicaca^30–32^.

The palaeohydrology of ancient lakes has played an essential role in the assembly of their endemic biota with significant lake-level fluctuations dramatically changing the outline, chemistry and ecological conditions of these basins^3,6^. The Titicaca has captured satellite lakes at higher altitudes in the Altiplano during major hydrological high stand phases of its geological history^32^. Extreme high water stands occurred during the Pleistocene (the palaeolake mentioned above Mataro c. 1.5–1.6 Ma) when the water level reached 140 m higher than at present, flooding most of the Altiplano^32^. Conversely, the Lake was significantly reduced as recently as ca. 90,000 years ago, when the water table presumably lowered to − 240 m, resulting in a water column of only 45 m at the deepest portions of the Chucuito basin. Other lake level regressions took place 12,000 years ago (− 110 m) and between 8000 and 3600 years ago, when the lake level was settled at − 90 m below its present stand^32^. These cycles of expansion and retreat, with the consequent changes in water salinity, may have exerted a major impact on the speciation and diversification of the Titicacan fauna^4,6,13–15^. The evolution of lacustrine species in the Altiplano may have been therefore governed by an intricate pattern of cycles of colonization followed by population expansions and intra-lacustrine diversification. In contrast, extinction and allopatric vicariance within sub-basins may have prevailed during regressions, with subsequent dispersal to different lakes and water streams of the Altiplano^14^.

### Morphological convergence and replicated radiations

The mitogenomic phylogeny agrees with previous results in uncovering a striking discordance with morphological taxonomic determinations, suggesting a high incidence of morphological convergence even among distant lineages^15^. This convergence is particularly notorious in generalist taxa with smooth body integument*,* that are polyphyletic as they show divergent mitogenomes placed in two or more distinct clades (e.g. in lineages A, B, C, D and E in the mitochondrial ML tree; see Fig. 2a). No significant association between morphology and nuclear phylogenetic relationships is detected either, suggesting that this pattern may fit better with replicated radiations rather than due to reticulate evolution^33^.

Habitat specialization and trophic regimes of Andean amphipods are mostly unknown. However, a considerable trophic overlap, omnivorous opportunist feeding habits and a high dispersal ability–such as those described elsewhere for the *Eulimnogammarus* amphipods of Lake Baikal^34^, could explain the high degree of convergence of generalist morphotypes observed in the Titicacan *Hyalella*. Besides, armoured spiny morphologies have evolved independently multiple times in the Titicacan *Hyalella*^15^, a result confirmed here with phylogenomic data as armoured body forms have episodically appeared in all Andean clades except in clade F (Figs. 2a,b and 3a). Spiny morphologies are frequent in several amphipod marine families. However, they are rare among epigean continental water forms, except for members of the Lake Baikal species-flock, with at least four independent transitions from non-spiny to spiny forms and two reversals^21^. Spines are also known in Caspian gammaroids, and in members of the genus *Fuxiana* (Lake Fuxian, China)^35,36^. Armoured spiny morphologies in the Titicaca have been related to the predation pressure exerted by the cyprinodontid killifish endemic to the lake^19,37^. Thus, these morphologies can be envisaged as the result of a coevolutionary arms race with predators in replicated radiations, resulting in convergence rather than representing key-innovation traits.

Notorious cases of replicated radiations leading to morphological convergence are known in ancient lacustrine systems, such as the cichlid fishes of Africa's rift lakes that radiated in parallel^40,41^ or the amphipod assemblage of Lake Baikal^21,42^. Trophic phenotypes of phylogenetically distinct clades of cichlids endemic to different lakes are textbook examples of morphological convergence^43^. This convergence derives from ecological opportunities and similar adaptive landscapes facilitating the evolution of similar suites of ecomorphs despite independent evolutionary histories^44^. Ecological opportunity, lack of competition and open habitats are factors that have been related to rapid diversification on islands and island-like systems such as lakes and mountains^45,46^. The ecological opportunities offered by the emergence of island-like habitats has been suggested as the leading cause of rapid diversification of *Lupinus* and other plant genera after the Andean uplift, with diversification rates not dissimilar to cichlid fish radiations in east African lakes^47^. Empty lacustrine habitats may have been repeatedly colonized by South American *Hyalella* linages after the Andean uplift and the formation of endorheic basins in the area, with the more successful generalist morphotypes rapidly diversifying in parallel.

### Concluding remarks

In summary, our results show that Andean amphipods derive from older South American lineages and have experienced recent diversification episodes linked to the uplift of the northern Andean Altiplano and the formation of high-altitude endorheic basins, which have suffered repeated cycles of expansion and retreat. The lack of resolution of the phylogenetic relationships among the different Altiplano *Hyalella* clades, the very shallow divergences displayed within most of the clades and the discordance between gene trees and morphology-based species denominations by the convergence of body forms, point to a diversification driven by the ecological opportunities offered by the island-like lacustrine habitats established after the Andean uplift. These results added to the recent developments accomplished in the study of evolutionary patterns of endemic Titicacan gastropods and fish^4,6,13^ establish an emergent study-system to understand species diversification.

## Methods

### Taxon sampling

We selected samples as a set of crucial representative taxa based on a *cox1* species delimitation analysis of material from Lake Titicaca and nearby high-altitude water bodies in Peru and Bolivia (see Jurado-Rivera et al.^15^). We also included congeneric taxa from southern Ecuador (high-altitude lakes at El Cajas Massif; Azuay) and southernmost Chile (Madre de Dios Island; Magallanes Region) for comparative purposes. A total 35 *Hyalella* specimens were used for this study, of which 19 collected in the Titicaca, 12 at other surrounding water bodies in the Altiplano, three from the Ecuador Andes and one from the Austral region of Chile (Table 1). Thirteen of the specimens fall within clade A of Adamowicz et al.^14^ and include four of the molecular operational taxonomic units delimited in Jurado-Rivera et al.^15^; other seven in clade D (representing two delimited spp.); seven in clade E (from four delimited spp.); three in clade C (MOTUs C1 and C2); and two in clade B (representing the delimited MOTU B1) (Table 1). Samples were collected using a hand-held plankton net or with a small dredge operated from the lakeshore or a boat. Specimens were preserved in the field in 96% ethanol immediately after collection. We also included in the analyses the mitogenomes of the Titicaca a species in a previous study^48^ and *H. azteca* retrieved from the genome project of this species (Bioproject accession PRJNA243935 *Hyalella azteca* U.S. Lab Strain^49^).

### Mitochondrial genomes

Genomic DNA from each sample was purified from a single specimen using the Qiagen DNeasy Blood & Tissue kit (Qiagen, Hilden, Germany) following manufacturer instructions and RNA was removed using 60 μg of RNAse A solution (Promega, Madison, WI, USA). Individual shotgun genomic libraries were constructed using the Hyper Library construction kit from Kapa Biosystems (Wilmington, Massachusetts, USA) from 100–500 ng of genomic DNA and fragments of about 480 pb that were indexed with Illumina TruSeq adapters. After quantification by qPCR, up to 13 libraries each corresponding to a particular sample/species were pooled in equimolar concentrations and pair-end sequenced (2 × 150 bp) in a single lane of Illumina HiSeq2500. Fastq files were demultiplexed with bcl2fastq software (Illumina) with adapter sequences and low-quality bases (< Q30) removed in Trimmomatic v0.33^50^. We assembled both paired and unpaired clean reads in SPAdes (v3.13)^51^ using three kmers (21, 35 and 47 nucleotides) to maximize assembly yield. We compared the contig sequences obtained in the previous step using BLASTn (e-value 30) against the mitogenome of *H. lucifugax* (ENA acc. number LT594767) as a reference to filter contigs containing mitochondrial sequences. The completion of the mitochondrial contigs was assessed using the script circularizationCheck.py (mitoMaker)^52^ (script available from https://github.com/RemiAllio/MitoFinder/blob/master/circularizationCheck.py) with contigs extended by mapping reads during several iteration steps in GENEIOUS 11.1.5 when needed^53^. The mitogenomes were annotated using MITOS2 webserver^54^ and genes manually curated in GENEIOUS, particularly at 5′ and 3′ ends.

### Ribosomal DNA sequences and nuclear single-copy orthologous genes

We used a similar BLASTn approach as described above to retrieve the contigs from each library matching the nuclear SSU and LSU ribosomal sequences of *H. azteca*^49^ and *Drosophila melanogaster* (GenBank acc. number M21017). To search for *Hyalella* single-copy nuclear orthologous genes we used Orthofinder v2.3.11^55^ to explore the contig sequences of each genome library to identify orthologous groups. First, all possible Open Reading Frames (ORFs) with a minimum length of 225 bp between stop codons at both strands for each contig sequence were identified from each *Hyalella* library using getorf (EMBOSS v6.6.0.0^56^). The obtained sequences were then translated into protein and the *Hyalella* specimen-specific protein-sequence files used as input for searching in Orthofinder using the dendroblast and diamond options. Subsequent analyses were based on the DNA version of the protein sequences found, and the results of a random species used to perform similarity searches against the *H. azteca* RefSeq genome database^49^ using tblastn to retrieve the corresponding orthologous sequences of the congeneric reference taxon.

### Phylogenetic analyses of mitochondrial genomes

Individual mitochondrial protein-coding genes (PCGs) were aligned at the amino-acid level using MUSCLE^57^ with the corresponding DNA sequences concatenated using Phyutility^58^. The best partition scheme starting from the 39 possible maximum (i.e. splitting by 13 PCGs and the three codon positions) and the best-fitting nucleotide substitution evolutionary models for each partition were estimated in IQ-TREE v1.6.10^59,60^. The partitioning scheme selected consisted in subdividing by codon position, with the exception that *atp8* 2nd sites that were included in the 1st position partition. The alternative simpler hypothesis consisting in including 2nd *atp8* sites in the corresponding 2nd partition retrieved a marginal larger BIC value (258,833.3737), so this option was selected for all posterior analyses. GTR + I + G4 was selected as the best substitution model for each mitogenome partition. The total cophenetic index of the phylogenetic tree obtained under the best partitioning scheme indicated that the tree is balanced (not asymmetrical; TCI = 2441, from a possible value range from 619 to 9139). For analyses in IQTREE we applied the parameter-rich “edge-unlinked partition model”, i.e. each partition has its own set of branch lengths, thus accounting for the possibility of a non-constant evolutionary rate through time for particular nucleotide positions (heterotachy). Nucleotide substitution saturation was explored using the Xia test in DAMBE6^61^ and the total cophenetic index to check the balance of the phylogenetic tree obtained under the best partitioning scheme^62^. Maximum Likelihood (ML) and Bayesian analyses were performed in IQ-TREE and MrBayes v3.2^63^, respectively. We also explored the implementation of codon-based substitution models^64^ through the analysis of the dataset at the protein level and using mixed models to accommodate data heterogeneity^63^. The mitogenomes of the distant amphipods *Parhyale hawaiensis* (NC_039402), *Platorchestia japonica* (MG010370) and *Platorchestia parapacifica* (MG010371) were included in the analyses and the former species used to root the trees. Resulting ML topologies were compared with the Approximately Unbiased Test of Phylogenetic Tree Selection as implemented in IQTREE.

### Phylogenetic analyses of nuclear ribosomal DNA sequences

We investigated the impact of considering ribosomal SSU and LSU secondary structures in both sequence alignments and phylogenetic hypotheses. First, we aligned *Hyalella* and outgroup sequences in MAFFT v7.450^65^ using the default FFT-NS-1 algorithm (i.e., without considering secondary structures). In other analyses, the secondary structures of the two nuclear ribosomal RNAs of *Anopheles albimanus* (L78065) were used as guides in RNAsalsa v0.8.1^66^ to define stem and loop regions at the highest stringency (i.e. 1.00), thus ensuring that only conserved loops were included in the alignment. We also analyzed ribosomal sequences partitioned independently as stems and loops to assess bias in nucleotide composition and substitutions rates^67^. The doublet model, i.e. assuming that base pairs in RNA secondary structures are not phylogenetically independent, was also explored in MrBayes. Finally, to assess the impact in the phylogenetic signal of both poorly aligned positions and highly divergent regions we examined the effect of not considering gaps using Gblocks v0.91b under default parameters^68^ or, alternatively, defined gaps as binary characters irrespective of their length^69^ in SeqState v1.4^70^. BIC scores indicated that a single partition merging the LSU and SSU sequences was preferred over other schemes such as considering the two genes as different partitions or partitioning by stems and loops, so the two ribosomal markers were concatenated for downstream phylogenetic analyses. ML and Bayesian analyses were performed as described above by implementing the substitution model selected (GTR + I + G4). The SSU and LSU sequences of *Parhyale hawaiensis* (obtained from its genome project Bioproject accession PRJNA306836^71^) and the SSU of *Platorchestia japonica* (EF582936.1) were used as outgroups. The *A. albimanus* guide sequences were removed from the final alignment.

### Phylogenetic analyses of nuclear single-copy orthologous genes

The orthologous gene regions sequenced in all samples were aligned using MAFFT with poorly aligned and gappy regions subsequently removed using the gappyout algorithm in trimAl v1.4^72^. Sliding windows of six bp were applied to identify regions showing divergences higher than 1.5× the average across the sequences using R and Perl scripts (available from https://github.com/brunonevado/trimming)^73^. These regions were considered as missing data. Furthermore, sequences shorter than 100 bp were excluded from the analysis after alignment. Individual ortholog alignments were concatenated in Phyutility and ML phylogeny was obtained using IQ-TREE implemented the best partition scheme (1st + 2nd codon sites GTR + I + G; 3rd sites GTR + G) with 1000 fast bootstrap support replicates. To explore congruence among individual gene trees and the concatenated alignment tree topology we performed the Shimodaira-Hasegawa test (SH test)^74^ using IQ-TREE v1.6.10. In addition, gene- and site-concordance factors^28^ calculating the proportion of genes (gCF) or nucleotide sites (sCF) that are concordant with any particular branch in the supermatrix (concatenated) tree were computed using IQ-TREE vs. 2.0.5^60^. As concatenation of genome DNA sequence data has been shown to be prone to systematic biases^75^ we also inferred the species trees based on the multispecies coalescent model (MSC). The StarBeast2 package^76^ in BEAST2 v. 2.6^77^ was used to implement the MSC using the 76 orthologous sequences. For this analysis, the 37 terminals were classified into 18 putative species after the results obtained in a previous molecular delimitation study using mitochondrial *cox1* sequences^15^. Bayesian analyses were run for 2 × 10^9^ generations sampling every 5000 using a single partition with a GTR + G model and the nucleotide substitution rate estimated from geological time constraints (see below). A strict clock was implemented for gene trees and a relaxed uncorrelated log-normal distribution prior for the species tree. The convergence of the runs and node age densities were assessed in Tracer v1.7.0. Species tree, gene trees, posterior probabilities and other parameters were estimated with the package Treeannotator included in BEAST2 distribution discarding the first 10% as burn-in. Another MSC approach was explored including the mitochondrial protein-coding genes as a single “gene” added to the 76 orthologous nuclear sequences assuming a 0.5 diploid value and appropriate model and substitution rates for the mitochondrial sequences. We also estimated the nuclear gene species tree in agreement with the largest number of quartet trees within a set of unrooted gene trees with ASTRAL III vs. 5.7.3^78^ . Comparison of mitochondrial and nuclear single-copy trees was based on the Baker’s Gamma correction^79^ , a measure of association between two dendrograms. To calculate the statistical significance of the index we performed a permutation test (100 replicates) by randomly shuffling the labels of one of the trees and calculating the distribution under the null hypothesis of fixed tree topologies.

### Estimation of divergence times

Molecular dating analyses were performed on the mitochondrial nucleotide sequence dataset after removing the divergent sequences of the outgroup *P. hawaiensis* and the two *Platorchestia* species. Topologies of the trees, model parameter values and node ages were co-estimated and optimized in BEAST v1.10.4^80^. Bayesian analyses were run for 100 million generations sampling every 10,000 generations. The convergence of the runs and node age densities were assessed in Tracer v1.7.0, and tree topology and parameters in TreeAnnotator. Clock models (strict vs uncorrelated log-normal), diversification models (Yule vs Birth–Death), and alternative tree topologies were compared based on Bayes Factors calculated with marginal likelihood values estimated from path-sampling analyses in BEAST (40 steps with 5 million generations each). As the reciprocal monophyly of North and South American *Hyalella* was not resolved in the previous analyses, we used Bayes Factors to contrast the two alternative hypotheses (e.g. *H. azteca* or South American clade C at the base of the tree). This analysis favoured the former hypothesis with moderate to strong support (Bayes Factor = 11.438). Consequently, for estimating *Hyalella* divergence times based on the mitogenome dataset in BEAST we used this tree topology as a constrain, with model parameter values and node ages optimized and co-estimated in the analysis from the data. A second Bayes Factor analysis selected a relaxed clock with an uncorrelated log-normal distribution even excluding the distant amphipod outgroups from the mitochondrial dataset (Bayes Factor = 55.8). The ages of two nodes were implemented separately as constraints to calibrate the relaxed molecular clock and compared by cross-validation. Each calibration point was specified as a log-normal distribution prior with 95% confidence intervals. Firstly, we assumed that the MRCA of clade A –a species group that likely derives from an intra-lacustrine diversification^14,15^– cannot precede the paleolake formation that gave rise to the present Lake Titicaca. The oldest and highest of the paleolakes in the Altiplano (Lake Mataro) lies just above an ash bed with an estimated age of 2.8 ± 0.4 Ma^30^. This age estimate and its confidence limits were used as age constraint for clade A. Alternatively, the age of the root of the tree was defined based on the *cox1* rate (0.0189 nucleotide substitutions per site per million years) obtained by Adamowicz et al.^14^ (20.63 Ma with a 95% confidence interval of 15.615–27.086 Ma). Mean values and confidence intervals of parameters and ages were estimated discarding 10% of the run as burn-in in both calibrations.

## Supplementary Information

Supplementary Information.

## Data Availability

The DNA sequences generated during the current study are available in GenBank with the following accession numbers: MT672015-MT672049 (mitochondrial genomes), MT823207-MT823242 (small subunit nuclear ribosomal sequences), MW047111-MW047146 (large subunit ribosomal nuclear sequences) and MW234509-MW237207; MW039618-MW039653 (single-copy orthologous nuclear sequences). The phylogenetic trees and DNA sequence alignments obtained in this study are available in nexus format in the GitHub repository (https://github.com/Frazapel/Hyalella-amphipod-Phylogenomics).
