# Supplementary material for: Phylogenomics of the Hyalella amphipod species-flock of the Andean Altiplano
Source: Sci Rep. 2021 Jan 11;11:366. doi: 10.1038/s41598-020-79620-4 (PMC7801522; doi:10.1038/s41598-020-79620-4)
Supplement: Supplementary file 1 — Supplementary Information. [file 41598_2020_79620_MOESM1_ESM.pdf]

Phylogenomics of the *Hyalella* amphipod species-flock of the Andean Altiplano

Francesco Zapelloni[1+], Joan Pons[2+], José A. Jurado-Rivera[1], Damià Jaume[2] & Carlos Juan[1,2,*]

[1] Dept. of Biology, University of the Balearic Islands. Ctra. Valldemossa km 7'5, Palma de Mallorca, 07122, Balearic Islands, Spain.

[2] IMEDEA (CSIC-UIB), Mediterranean Institute for Advanced Studies. C/ Miquel Marquès 21, Esporles, 07190, Balearic Islands, Spain.

* Correspondence author: cjuan@uib.es
[+] These authors contributed equally to this work

Supplementary Text 1.

Details of the mitochondrial genomes. Sixteen of the mitogenomes were complete (i.e. circular sequences) or almost complete rendering sizes ranging from 14.8 to 15.7 Kb. For uncomplete mitogenomes the control region could not be confidently assembled due to lack of sequence coverage, the presence of large poly-A runs or the presence of repeats longer than reads lengths. For the remaining mitogenome regions, some of the tRNA genes or segments of the protein-coding genes (PCGs) nad4, nad5 or nad6 were not present in the libraries so their sequences could not be obtained (Supplementary Table 1). Completed mitogenomes included the standard 13 PCGs, 22 transfer RNAs (tRNAs) and two ribosomal (rRNAs) genes displaying a PCG order matching the ancestral putative pancrustacean mitogenome, but some tRNA genes displayed rearrangements respect to the order of other crustaceans as noticed elsewhere (Juan et al. 2016). The *Hyalella* mitogenomes showed an average 68.9% A + T content for the complete mitogenome sequences (Supplementary Table 1).

Xia's saturation test of the mitochondrial sequence dataset. Xia's test (Xia 217) showed low levels of substitution saturation in these sequences under the assumption of a symmetric tree (ISS = 0.339, ISSc = 0.572, P two-tailed <0.00001). Similar results were found when testing for saturation at each codon position separately, but for third coding positions moderate levels of saturation were detected (ISS = 0.606, ISSc = 0.554, P two-tailed <0.00001).

Single-copy nuclear gene fragment dataset. A total of 76 single-copy nuclear gene segments present in the 36 low-coverage *Hyalella* genomic libraries were retrieved using Orthofinder (Emms & Kelly, 2019.. BLAST similarity searches showed that 53 orthologous gene fragments matched known or predicted proteins of *H. azteca* (Supplementary Table 3). The sequences of several genome libraries corresponding to a particular ortholog were discarded either due to their short length or because they displayed an unusually high genetic divergence respect to the average across samples. Individual gene fragment alignments had a mean of 455 bp after trimming (from a minimum of 222 to a maximum of 1,116 bp; 132 bp SD) with 95 bp parsimony-informative positions on average (29 - 278 bp; 46 bp SD) (see Supplementary Table 3).

Juan, C., Jurado-Rivera J. A, Moreno, E Wolf, C., Jaume, D. & Pons, J. The mitogenome of the amphipod *Hyalella lucifugax* (Crustacea) and its phylogenetic placement. *Mitochondrial DNA Part B* 1, 755–756 (2016).

Emms, D. M. & Kelly, S. OrthoFinder: Phylogenetic orthology inference for comparative genomics. *Genome Biol.* 20, 238 (2019).

Xia, X. DAMBE6: New tools for microbial genomics, phylogenetics, and molecular evolution. *J. Hered.* 108, 431–437 (2017).

Supplementary Figure 1. Phylogenetic trees based on the 13 mitochondrial protein-coding genes (PCGs) under both Maximum Likelihood (ML) and Bayesian frameworks at both nucleotide (nt) and amino acid levels implementing various nt and codon substitution models (a-j). Circles in green indicate maximum nodal support. ML tree obtained with IQ-TREE using the best nt single partition model (a). MrBayes tree under a single nt partition with a mixed+I+G model (b). MrBayes tree using the codons model with codons equal (c), with model GY94 (d) and model M3 (e). ML amino acid tree (IQ-TREE) with model mt-MET+I+F+G4 (f). MrBayes tree based on amino acid data and the Mixed+G model (g), model mtZOA (h) and the mtMet+I+G model (i) PhyloBayes (Lartillot and Philippe, 2004;2005) amino acid tree under a mixed model CARgtr (j).

Lartillot, N. & Philippe, H. A Bayesian Mixture Model for Across-Site Heterogeneities in the Amino-Acid Replacement Process. *Mol. Biol. Evol.* **21**, 1095-1109 (2004).

Lartillot, N. & Philippe, H. Computing Bayes Factors using Thermodynamic Integration. Syst. Biol. 55, 195-207 (2005).

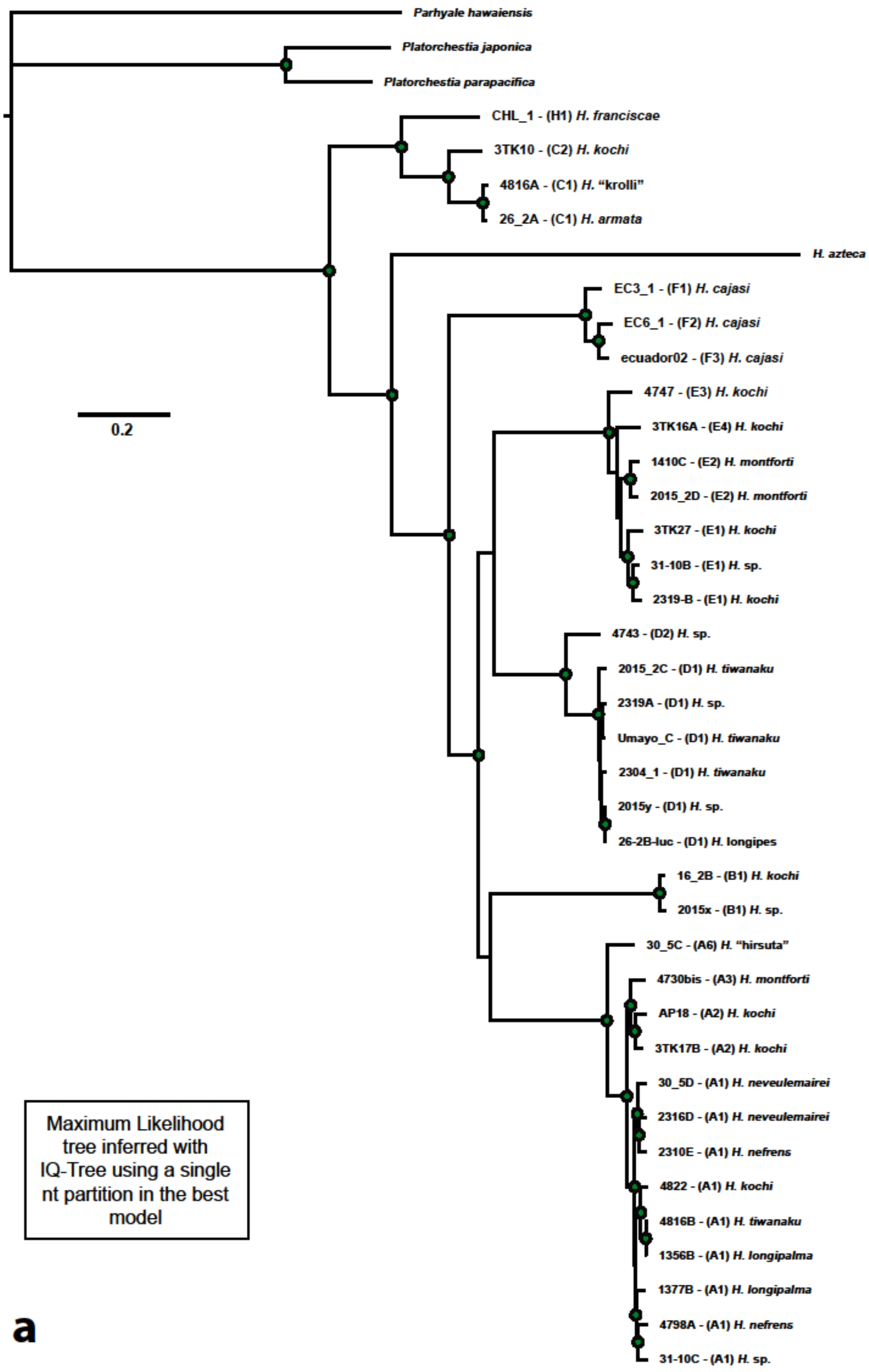

Parhyale hawaiensis
Platorchestia japonica
Platorchestia parapacifica
CHL_1 - (H1) H. franciscae
3TK10 - (C2) H. kochi
4816A - (C1) H. “krolli”
26_2A - (C1) H. armata
H. azteca
EC3_1 - (F1) H. cajasi
EC6_1 - (F2) H. cajasi
ecuador02 - (F3) H. cajasi
4747 - (E3) H. kochi
3TK16A - (E4) H. kochi
1410C - (E2) H. montforti
2015_2D - (E2) H. montforti
3TK27 - (E1) H. kochi
31-10B - (E1) H. sp.
2319-B - (E1) H. kochi
4743 - (D2) H. sp.
2015_2C - (D1) H. tiwanaku
2319A - (D1) H. sp.
Umayo_C - (D1) H. tiwanaku
2304_1 - (D1) H. tiwanaku
2015y - (D1) H. sp.
26-2B-luc - (D1) H. longipes
16_2B - (B1) H. kochi
2015x - (B1) H. sp.
30_5C - (A6) H. “hirsuta”
4730bis - (A3) H. montforti
AP18 - (A2) H. kochi
3TK17B - (A2) H. kochi
30_5D - (A1) H. neveulemairei
2316D - (A1) H. neveulemairei
2310E - (A1) H. nefrens
4822 - (A1) H. kochi
4816B - (A1) H. tiwanaku
1356B - (A1) H. longipalma
1377B - (A1) H. longipalma
4798A - (A1) H. nefrens
31-10C - (A1) H. sp.
0.2
Maximum Likelihood tree inferred with IQ-Tree using a single nt partition in the best model
a

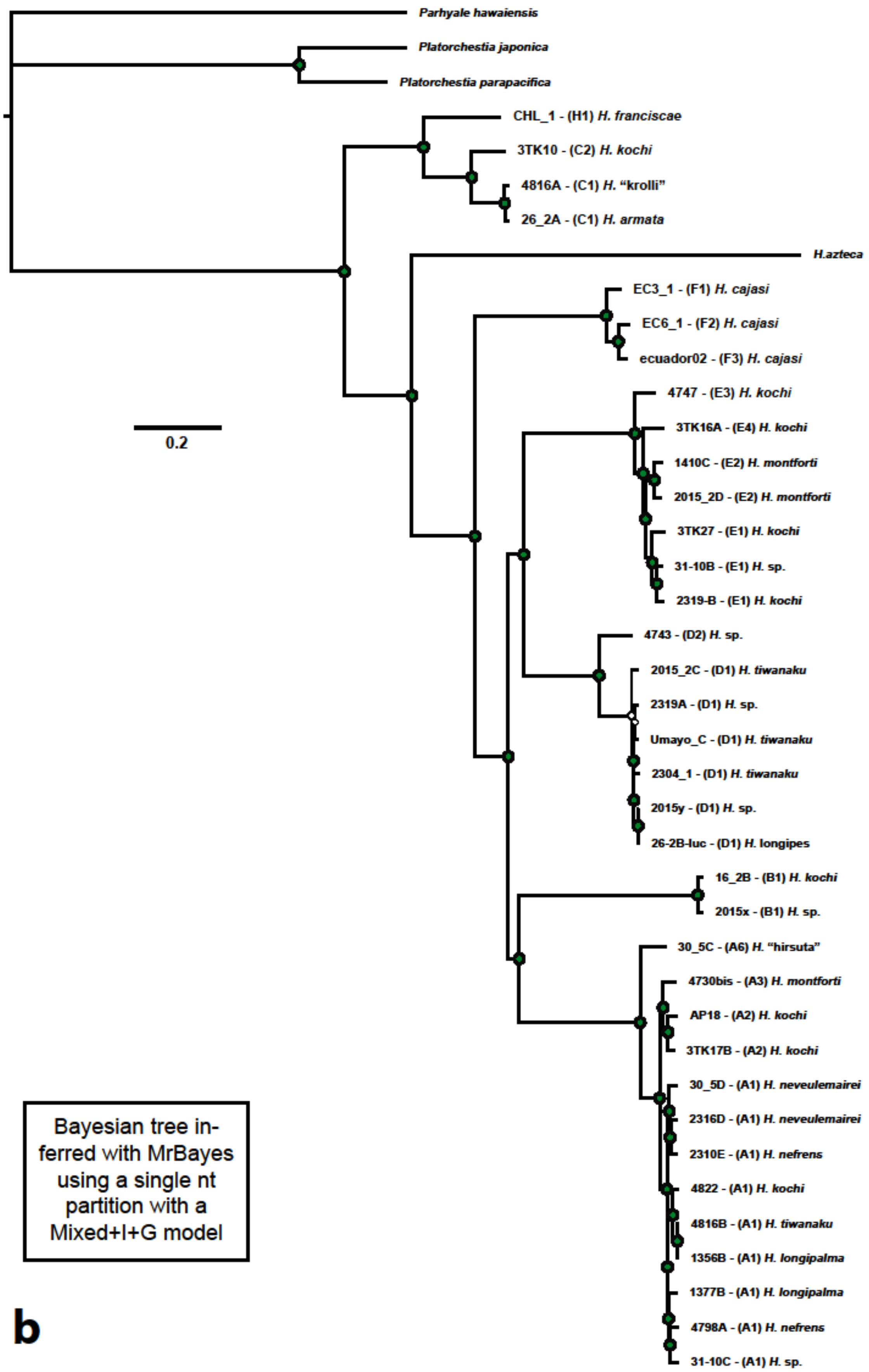

Bayesian tree inferred with MrBayes using a single nt partition with a Mixed+I+G model

b

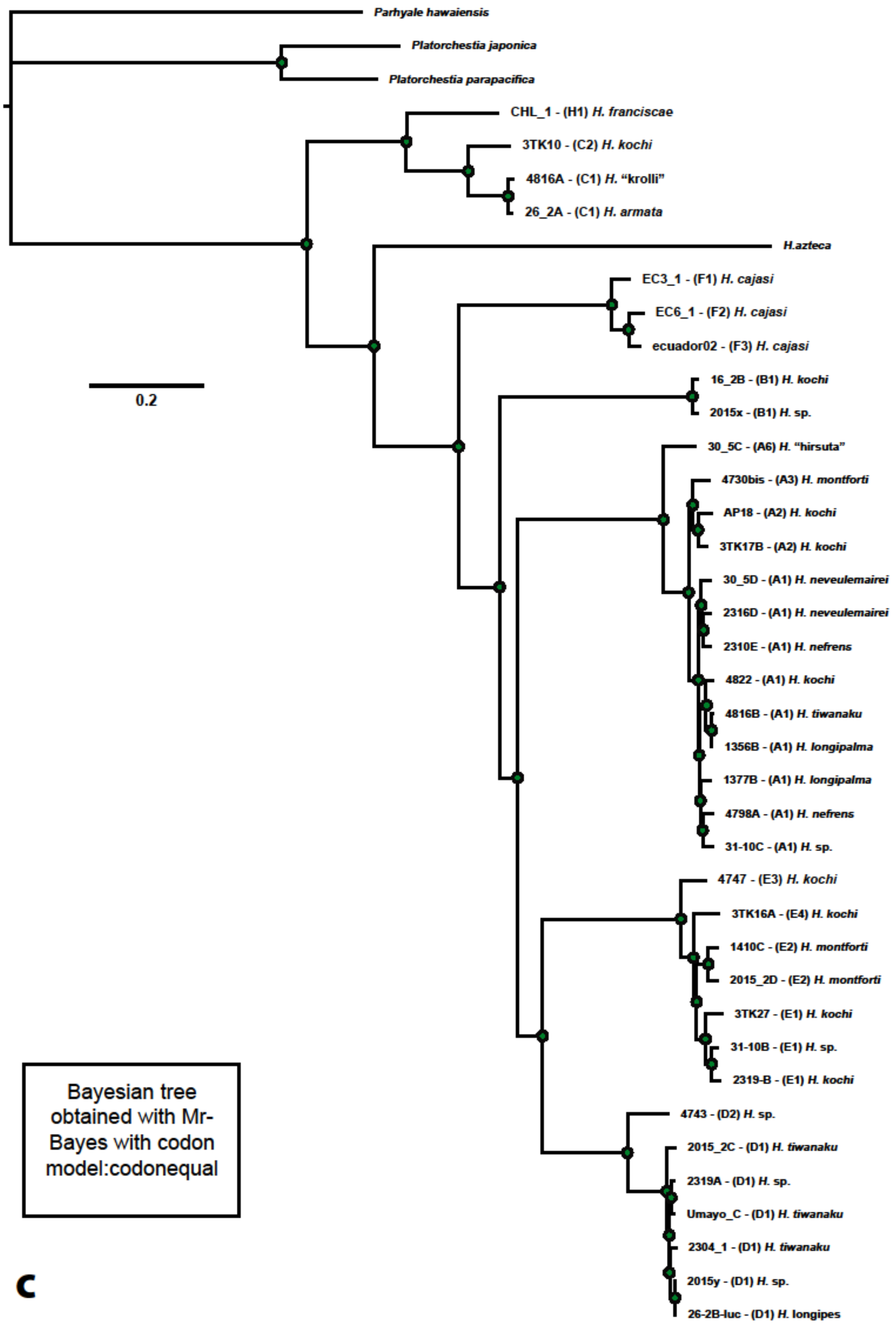

Parhyale hawaiensis
Platorchestia japonica
Platorchestia parapacifica
CHL_1 - (H1) H. franciscae
3TK10 - (C2) H. kochi
4816A - (C1) H. "krolli"
26_2A - (C1) H. armata
H.azteca
EC3_1 - (F1) H. cajasi
EC6_1 - (F2) H. cajasi
ecuador02 - (F3) H. cajasi
0.2
16_2B - (B1) H. kochi
2015x - (B1) H. sp.
30_5C - (A6) H. "hirsuta"
4730bis - (A3) H. montforti
AP18 - (A2) H. kochi
3TK17B - (A2) H. kochi
30_5D - (A1) H. neveulemairei
2316D - (A1) H. neveulemairei
2310E - (A1) H. nefrens
4822 - (A1) H. kochi
4816B - (A1) H. tiwanaku
1356B - (A1) H. longipalma
1377B - (A1) H. longipalma
4798A - (A1) H. nefrens
31-10C - (A1) H. sp.
4747 - (E3) H. kochi
3TK16A - (E4) H. kochi
1410C - (E2) H. montforti
2015_2D - (E2) H. montforti
3TK27 - (E1) H. kochi
31-10B - (E1) H. sp.
2319-B - (E1) H. kochi
Bayesian tree obtained with Mr-Bayes with codon model:codonequal
4743 - (D2) H. sp.
2015_2C - (D1) H. tiwanaku
2319A - (D1) H. sp.
Umayo_C - (D1) H. tiwanaku
2304_1 - (D1) H. tiwanaku
2015y - (D1) H. sp.
26-2B-luc - (D1) H. longipes
c

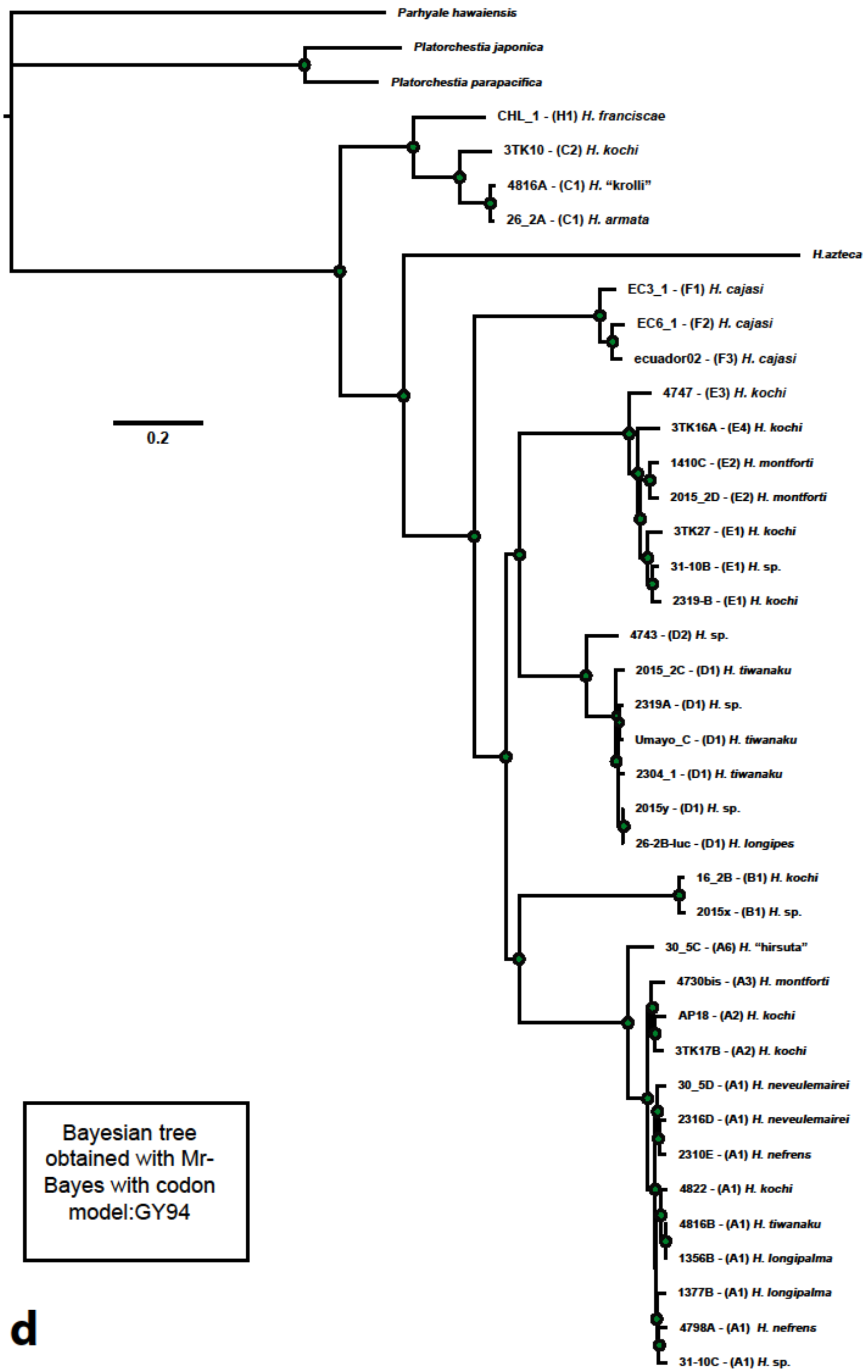
Parhyale hawaiensis
Platorchestia japonica
Platorchestia parapacifica
CHL_1 - (H1) H. franciscae
3TK10 - (C2) H. kochi
4816A - (C1) H. "krolli"
26_2A - (C1) H. armata
H.azteca
EC3_1 - (F1) H. cajasi
EC6_1 - (F2) H. cajasi
ecuador02 - (F3) H. cajasi
4747 - (E3) H. kochi
3TK16A - (E4) H. kochi
1410C - (E2) H. montforti
2015_2D - (E2) H. montforti
3TK27 - (E1) H. kochi
31-10B - (E1) H. sp.
2319-B - (E1) H. kochi
4743 - (D2) H. sp.
2015_2C - (D1) H. tiwanaku
2319A - (D1) H. sp.
Umayo_C - (D1) H. tiwanaku
2304_1 - (D1) H. tiwanaku
2015y - (D1) H. sp.
26-2B-luc - (D1) H. longipes
16_2B - (B1) H. kochi
2015x - (B1) H. sp.
30_5C - (A6) H. "hirsuta"
4730bis - (A3) H. montforti
AP18 - (A2) H. kochi
3TK17B - (A2) H. kochi
30_5D - (A1) H. neveulemairei
2316D - (A1) H. neveulemairei
2310E - (A1) H. nefrens
4822 - (A1) H. kochi
4816B - (A1) H. tiwanaku
1356B - (A1) H. longipalma
1377B - (A1) H. longipalma
4798A - (A1) H. nefrens
31-10C - (A1) H. sp.
0.2
Bayesian tree obtained with Mr-Bayes with codon model:GY94
d

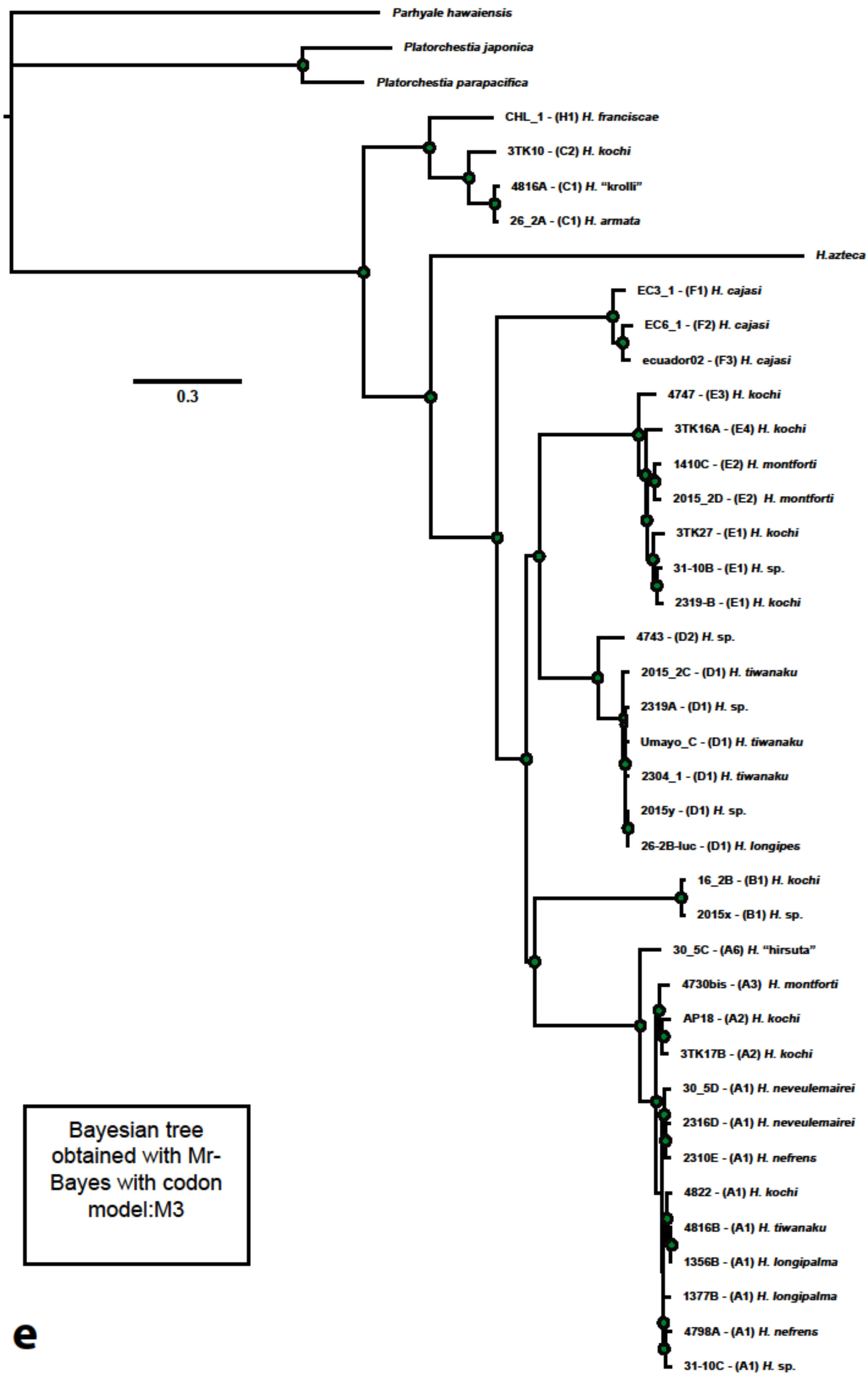

Parhyale hawaiensis
Platorchestia japonica
Platorchestia parapacifica
CHL_1 - (H1) H. franciscae
3TK10 - (C2) H. kochi
4816A - (C1) H. "krolli"
26_2A - (C1) H. armata
H.azteca
EC3_1 - (F1) H. cajasi
EC6_1 - (F2) H. cajasi
ecuador02 - (F3) H. cajasi
0.3
4747 - (E3) H. kochi
3TK16A - (E4) H. kochi
1410C - (E2) H. montforti
2015_2D - (E2) H. montforti
3TK27 - (E1) H. kochi
31-10B - (E1) H. sp.
2319-B - (E1) H. kochi
4743 - (D2) H. sp.
2015_2C - (D1) H. tiwanaku
2319A - (D1) H. sp.
Umayo_C - (D1) H. tiwanaku
2304_1 - (D1) H. tiwanaku
2015y - (D1) H. sp.
26-2B-luc - (D1) H. longipes
16_2B - (B1) H. kochi
2015x - (B1) H. sp.
30_5C - (A6) H. "hirsuta"
4730bis - (A3) H. montforti
AP18 - (A2) H. kochi
3TK17B - (A2) H. kochi
30_5D - (A1) H. neveulemairei
2316D - (A1) H. neveulemairei
2310E - (A1) H. nefrens
4822 - (A1) H. kochi
4816B - (A1) H. tiwanaku
1356B - (A1) H. longipalma
1377B - (A1) H. longipalma
4798A - (A1) H. nefrens
31-10C - (A1) H. sp.
Bayesian tree obtained with Mr-Bayes with codon model:M3
e

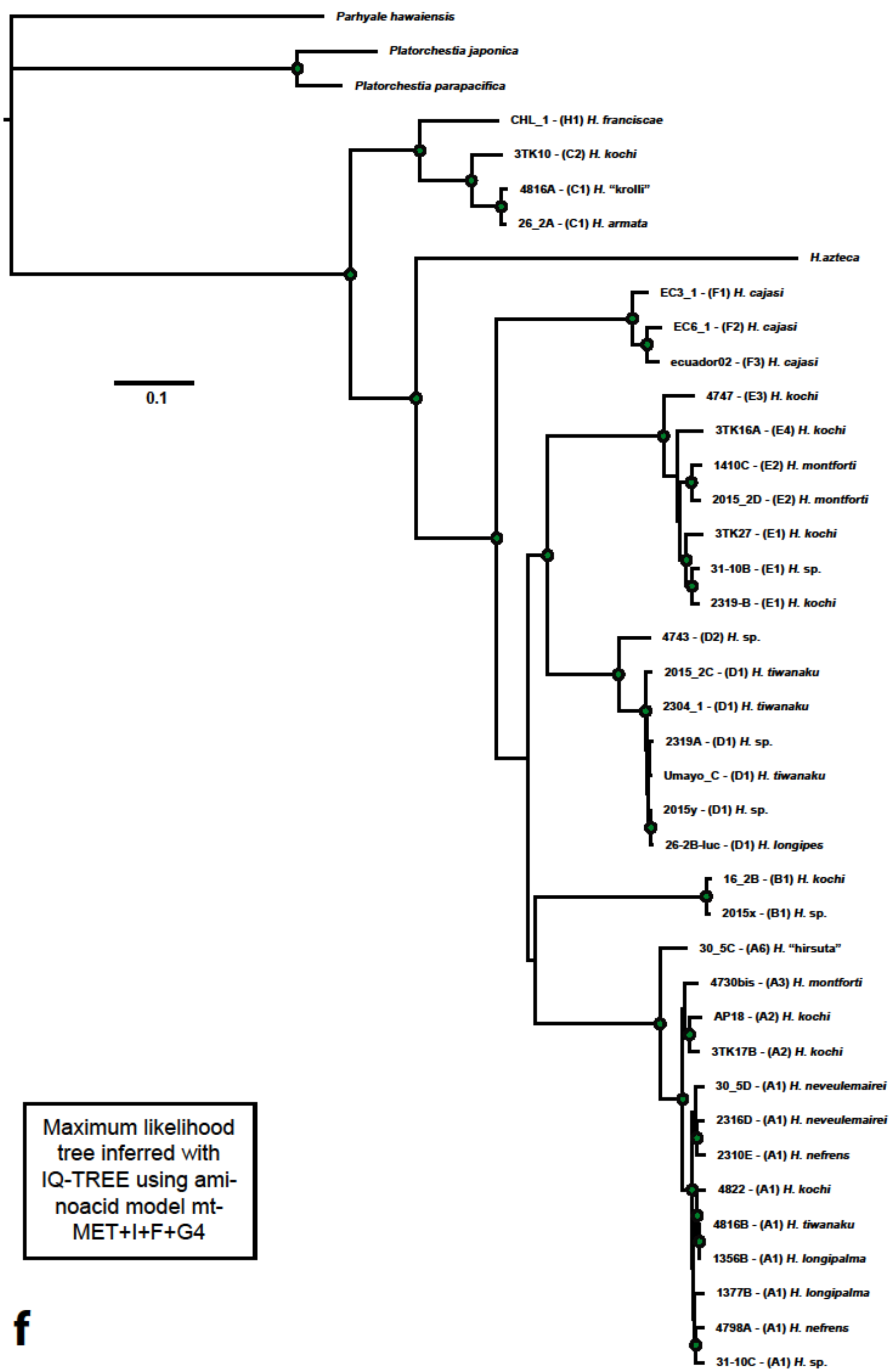
Parhyale hawaiensis
Platorchestia japonica
Platorchestia parapacifica
CHL_1 - (H1) H. franciscae
3TK10 - (C2) H. kochi
4816A - (C1) H. "krolli"
26_2A - (C1) H. armata
H.azteca
EC3_1 - (F1) H. cajasi
EC6_1 - (F2) H. cajasi
ecuador02 - (F3) H. cajasi
0.1
4747 - (E3) H. kochi
3TK16A - (E4) H. kochi
1410C - (E2) H. montforti
2015_2D - (E2) H. montforti
3TK27 - (E1) H. kochi
31-10B - (E1) H. sp.
2319-B - (E1) H. kochi
4743 - (D2) H. sp.
2015_2C - (D1) H. tiwanaku
2304_1 - (D1) H. tiwanaku
2319A - (D1) H. sp.
Umayo_C - (D1) H. tiwanaku
2015y - (D1) H. sp.
26-2B-luc - (D1) H. longipes
16_2B - (B1) H. kochi
2015x - (B1) H. sp.
30_5C - (A6) H. "hirsuta"
4730bis - (A3) H. montforti
AP18 - (A2) H. kochi
3TK17B - (A2) H. kochi
30_5D - (A1) H. neveulemairei
2316D - (A1) H. neveulemairei
2310E - (A1) H. nefrens
4822 - (A1) H. kochi
4816B - (A1) H. tiwanaku
1356B - (A1) H. longipalma
1377B - (A1) H. longipalma
4798A - (A1) H. nefrens
31-10C - (A1) H. sp.
Maximum likelihood tree inferred with IQ-TREE using ami-noacid model mt-MET+I+F+G4

f

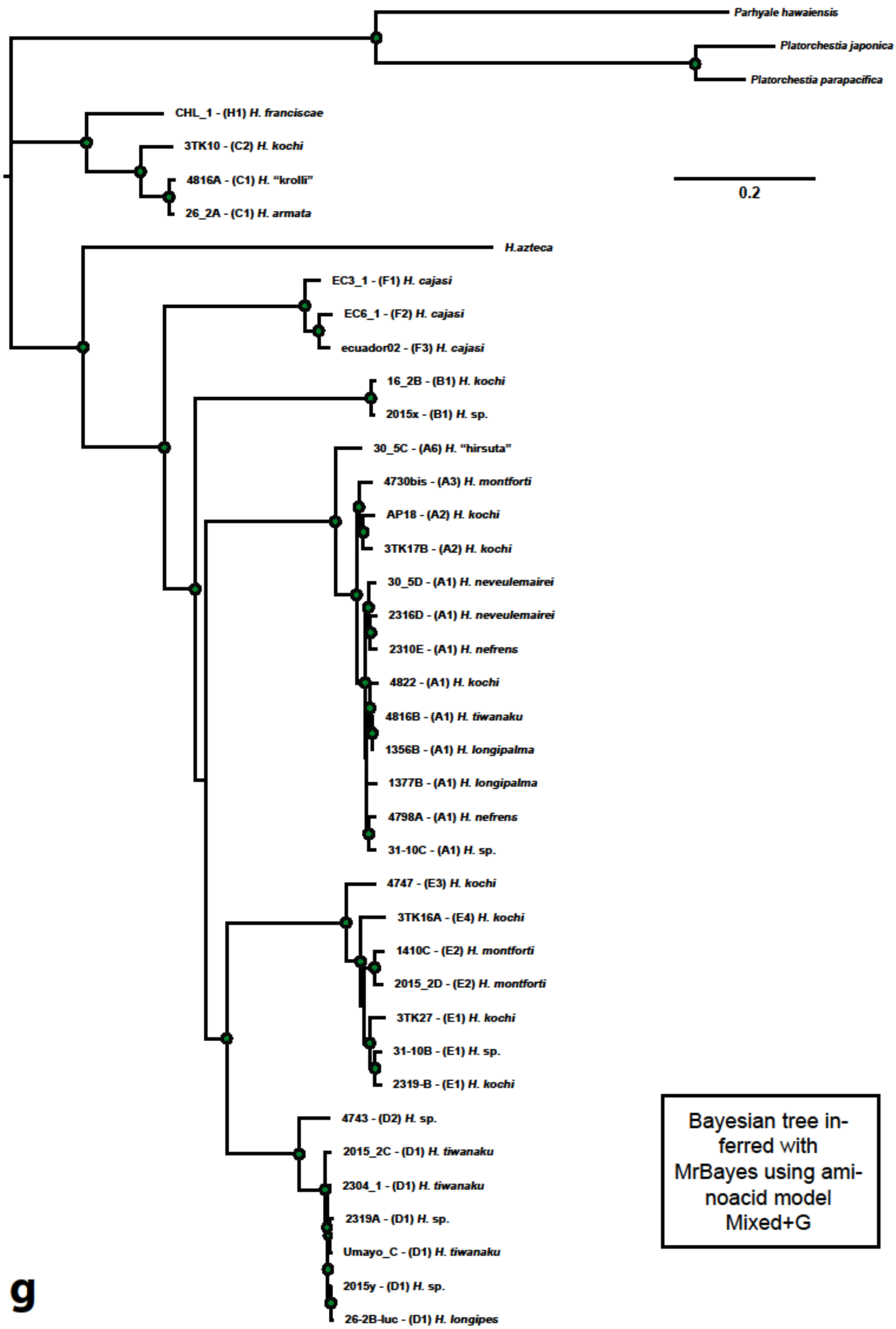
Parhyale hawaiensis
Platorchestia japonica
Platorchestia parapacifica
CHL_1 - (H1) H. franciscae
3TK10 - (C2) H. kochi
4816A - (C1) H. "krolli"
26_2A - (C1) H. armata
0.2
H.azteca
EC3_1 - (F1) H. cajasi
EC6_1 - (F2) H. cajasi
ecuador02 - (F3) H. cajasi
16_2B - (B1) H. kochi
2015x - (B1) H. sp.
30_5C - (A6) H. "hirsuta"
4730bis - (A3) H. montforti
AP18 - (A2) H. kochi
3TK17B - (A2) H. kochi
30_5D - (A1) H. neveulemairei
2316D - (A1) H. neveulemairei
2310E - (A1) H. nefrens
4822 - (A1) H. kochi
4816B - (A1) H. tiwanaku
1356B - (A1) H. longipalma
1377B - (A1) H. longipalma
4798A - (A1) H. nefrens
31-10C - (A1) H. sp.
4747 - (E3) H. kochi
3TK16A - (E4) H. kochi
1410C - (E2) H. montforti
2015_2D - (E2) H. montforti
3TK27 - (E1) H. kochi
31-10B - (E1) H. sp.
2319-B - (E1) H. kochi
4743 - (D2) H. sp.
2015_2C - (D1) H. tiwanaku
2304_1 - (D1) H. tiwanaku
2319A - (D1) H. sp.
Umayo_C - (D1) H. tiwanaku
2015y - (D1) H. sp.
26-2B-luc - (D1) H. longipes
Bayesian tree inferred with MrBayes using aminoacid model Mixed+G
g

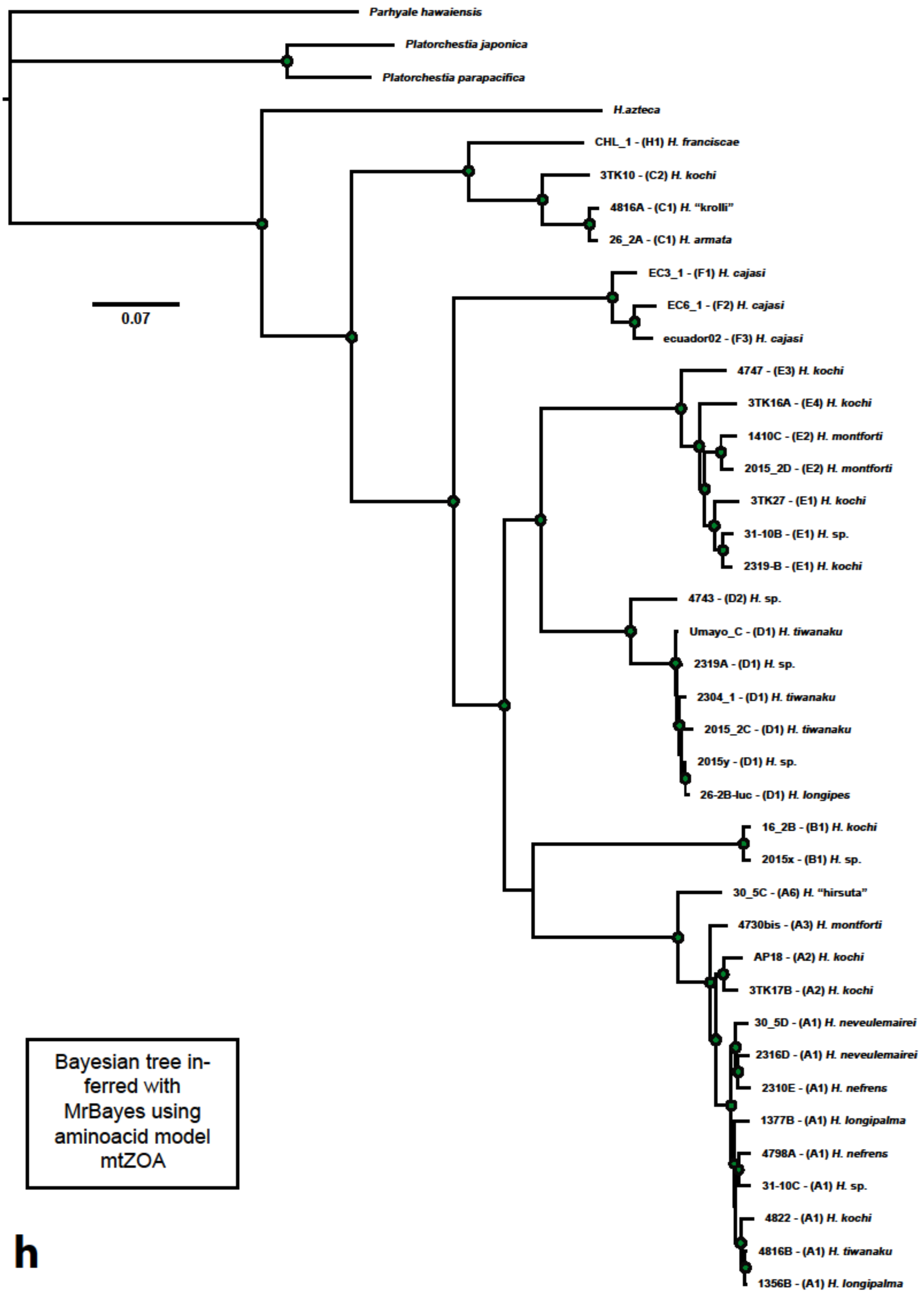

Parhyale hawaiensis
Platorchestia japonica
Platorchestia parapacifica
H.azteca
CHL_1 - (H1) H. franciscae
3TK10 - (C2) H. kochi
4816A - (C1) H. "krolli"
26_2A - (C1) H. armata
EC3_1 - (F1) H. cajasi
EC6_1 - (F2) H. cajasi
ecuador02 - (F3) H. cajasi
0.07
4747 - (E3) H. kochi
3TK16A - (E4) H. kochi
1410C - (E2) H. montforti
2015_2D - (E2) H. montforti
3TK27 - (E1) H. kochi
31-10B - (E1) H. sp.
2319-B - (E1) H. kochi
4743 - (D2) H. sp.
Umayo_C - (D1) H. tiwanaku
2319A - (D1) H. sp.
2304_1 - (D1) H. tiwanaku
2015_2C - (D1) H. tiwanaku
2015y - (D1) H. sp.
26-2B-luc - (D1) H. longipes
16_2B - (B1) H. kochi
2015x - (B1) H. sp.
30_5C - (A6) H. "hirsuta"
4730bis - (A3) H. montforti
AP18 - (A2) H. kochi
3TK17B - (A2) H. kochi
30_5D - (A1) H. neveulemairei
2316D - (A1) H. neveulemairei
2310E - (A1) H. nefrens
1377B - (A1) H. longipalma
4798A - (A1) H. nefrens
31-10C - (A1) H. sp.
4822 - (A1) H. kochi
4816B - (A1) H. tiwanaku
1356B - (A1) H. longipalma
Bayesian tree inferred with MrBayes using aminoacid model mtZOA

h

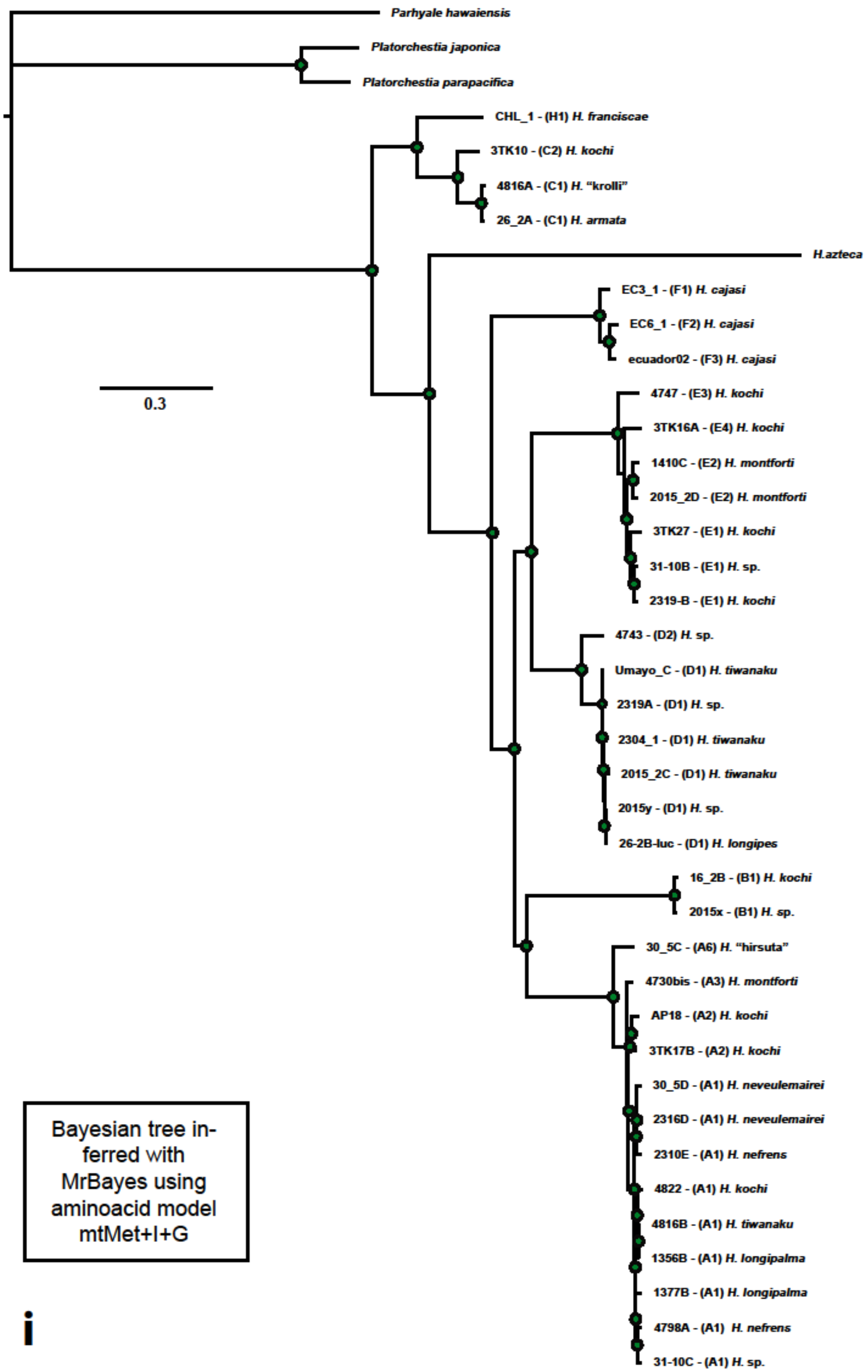
Parhyale hawaiensis
Platorchestia japonica
Platorchestia parapacifica
CHL_1 - (H1) H. franciscae
3TK10 - (C2) H. kochi
4816A - (C1) H. "krolli"
26_2A - (C1) H. armata
H.azteca
EC3_1 - (F1) H. cajasi
EC6_1 - (F2) H. cajasi
ecuador02 - (F3) H. cajasi
0.3
4747 - (E3) H. kochi
3TK16A - (E4) H. kochi
1410C - (E2) H. montforti
2015_2D - (E2) H. montforti
3TK27 - (E1) H. kochi
31-10B - (E1) H. sp.
2319-B - (E1) H. kochi
4743 - (D2) H. sp.
Umayo_C - (D1) H. tiwanaku
2319A - (D1) H. sp.
2304_1 - (D1) H. tiwanaku
2015_2C - (D1) H. tiwanaku
2015y - (D1) H. sp.
26-2B-luc - (D1) H. longipes
16_2B - (B1) H. kochi
2015x - (B1) H. sp.
30_5C - (A6) H. "hirsuta"
4730bis - (A3) H. montforti
AP18 - (A2) H. kochi
3TK17B - (A2) H. kochi
30_5D - (A1) H. neveulemairei
2316D - (A1) H. neveulemairei
2310E - (A1) H. nefrens
4822 - (A1) H. kochi
4816B - (A1) H. tiwanaku
1356B - (A1) H. longipalma
1377B - (A1) H. longipalma
4798A - (A1) H. nefrens
31-10C - (A1) H. sp.
Bayesian tree inferred with MrBayes using aminoacid model mtMet+I+G
i

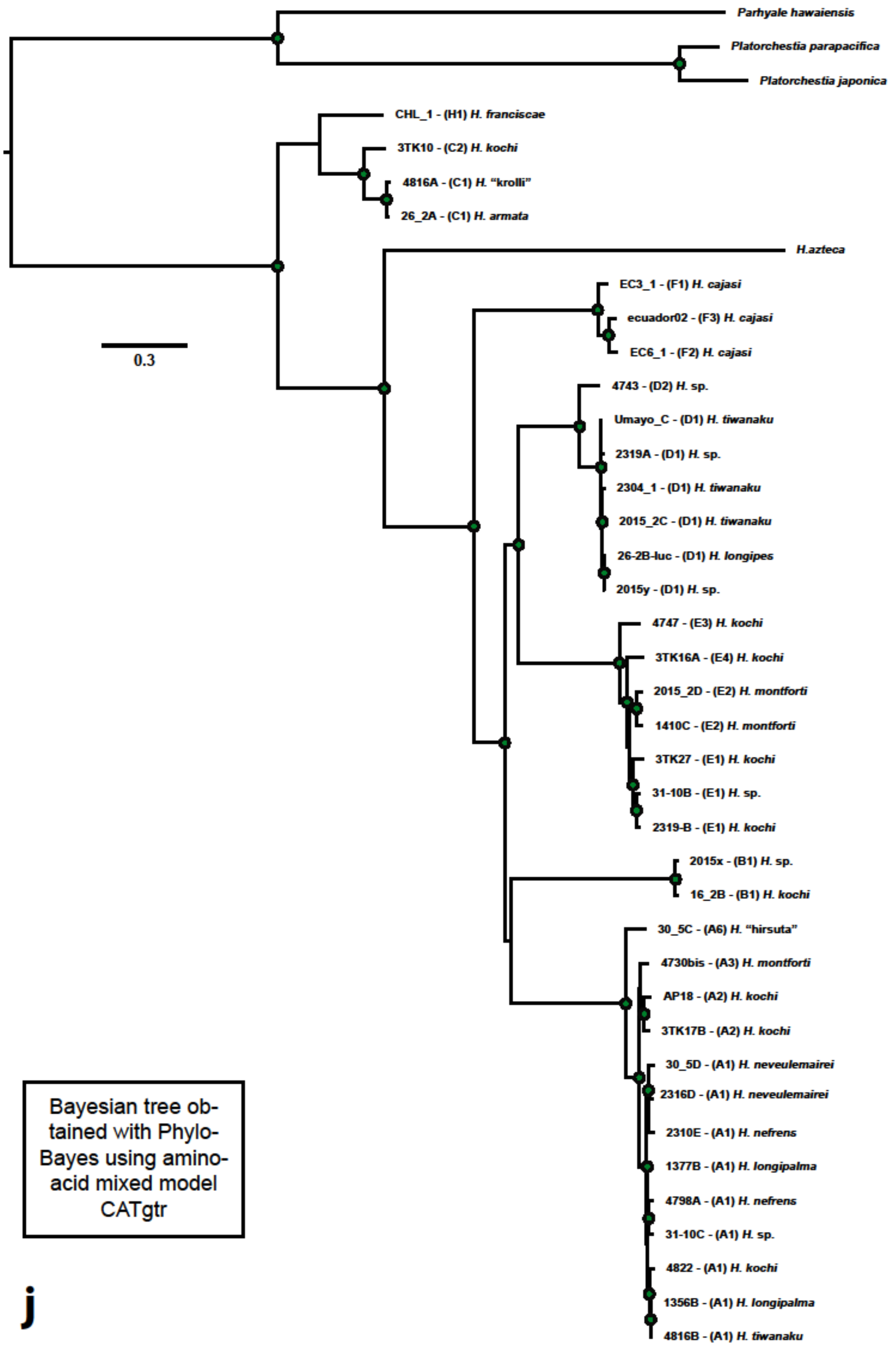
Parhyale hawaiensis
Platorchestia parapacifica
Platorchestia japonica
CHL_1 - (H1) H. franciscae
3TK10 - (C2) H. kochi
4816A - (C1) H. “krolli”
26_2A - (C1) H. armata
H.azteca
EC3_1 - (F1) H. cajasi
ecuador02 - (F3) H. cajasi
EC6_1 - (F2) H. cajasi
0.3
4743 - (D2) H. sp.
Umayo_C - (D1) H. tiwanaku
2319A - (D1) H. sp.
2304_1 - (D1) H. tiwanaku
2015_2C - (D1) H. tiwanaku
26-2B-luc - (D1) H. longipes
2015y - (D1) H. sp.
4747 - (E3) H. kochi
3TK16A - (E4) H. kochi
2015_2D - (E2) H. montforti
1410C - (E2) H. montforti
3TK27 - (E1) H. kochi
31-10B - (E1) H. sp.
2319-B - (E1) H. kochi
2015x - (B1) H. sp.
16_2B - (B1) H. kochi
30_5C - (A6) H. “hirsuta”
4730bis - (A3) H. montforti
AP18 - (A2) H. kochi
3TK17B - (A2) H. kochi
30_5D - (A1) H. neveulemairei
2316D - (A1) H. neveulemairei
2310E - (A1) H. nefrens
1377B - (A1) H. longipalma
4798A - (A1) H. nefrens
31-10C - (A1) H. sp.
4822 - (A1) H. kochi
1356B - (A1) H. longipalma
4816B - (A1) H. tiwanaku
Bayesian tree obtained with PhyloBayes using amino-acid mixed model CATgtr
j

Supplementary Figure 2. Maximum Likelihood trees based on nine single mitochondrial PCGs. Phylogenies were inferred using IQ-TREE. The partitions were determined by codon positions, the best model for each partition was obtained with ModelFinder.

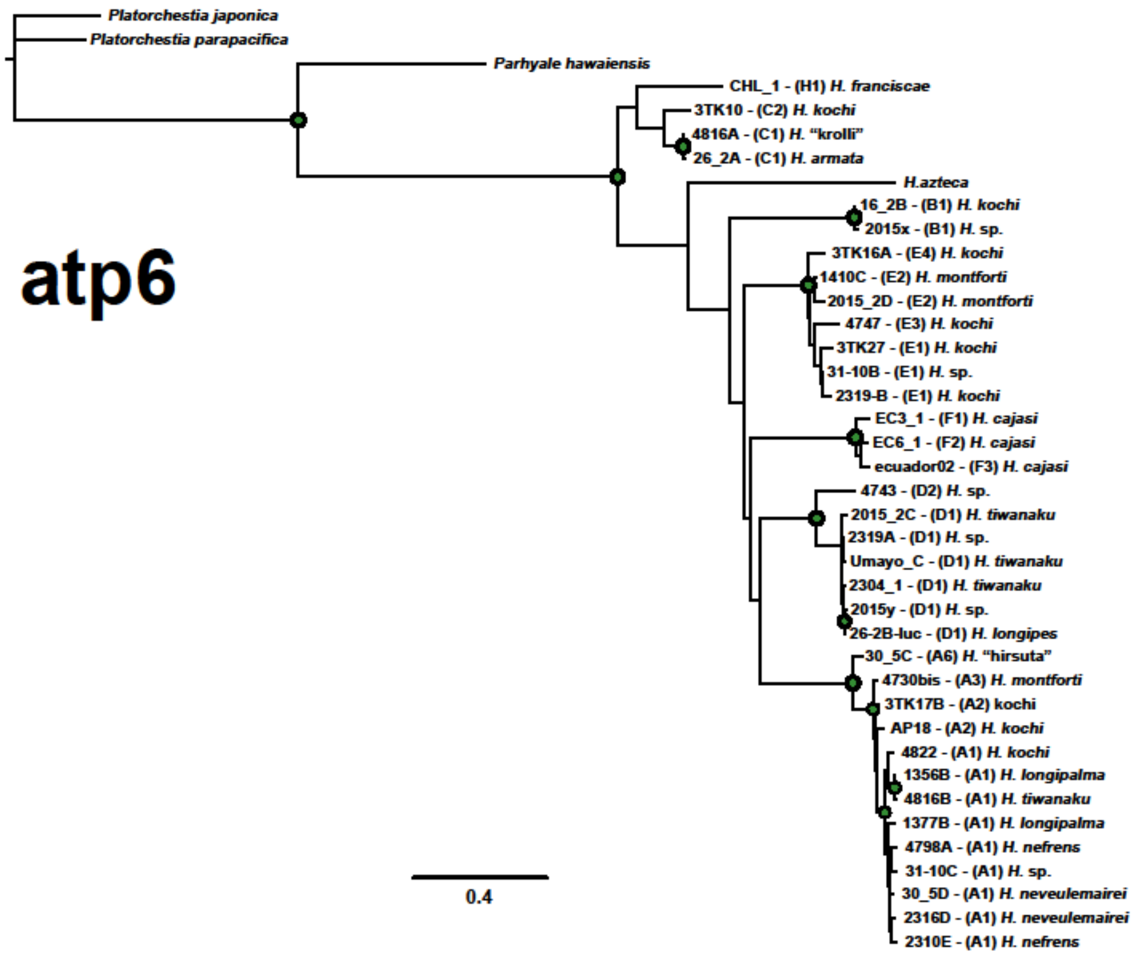

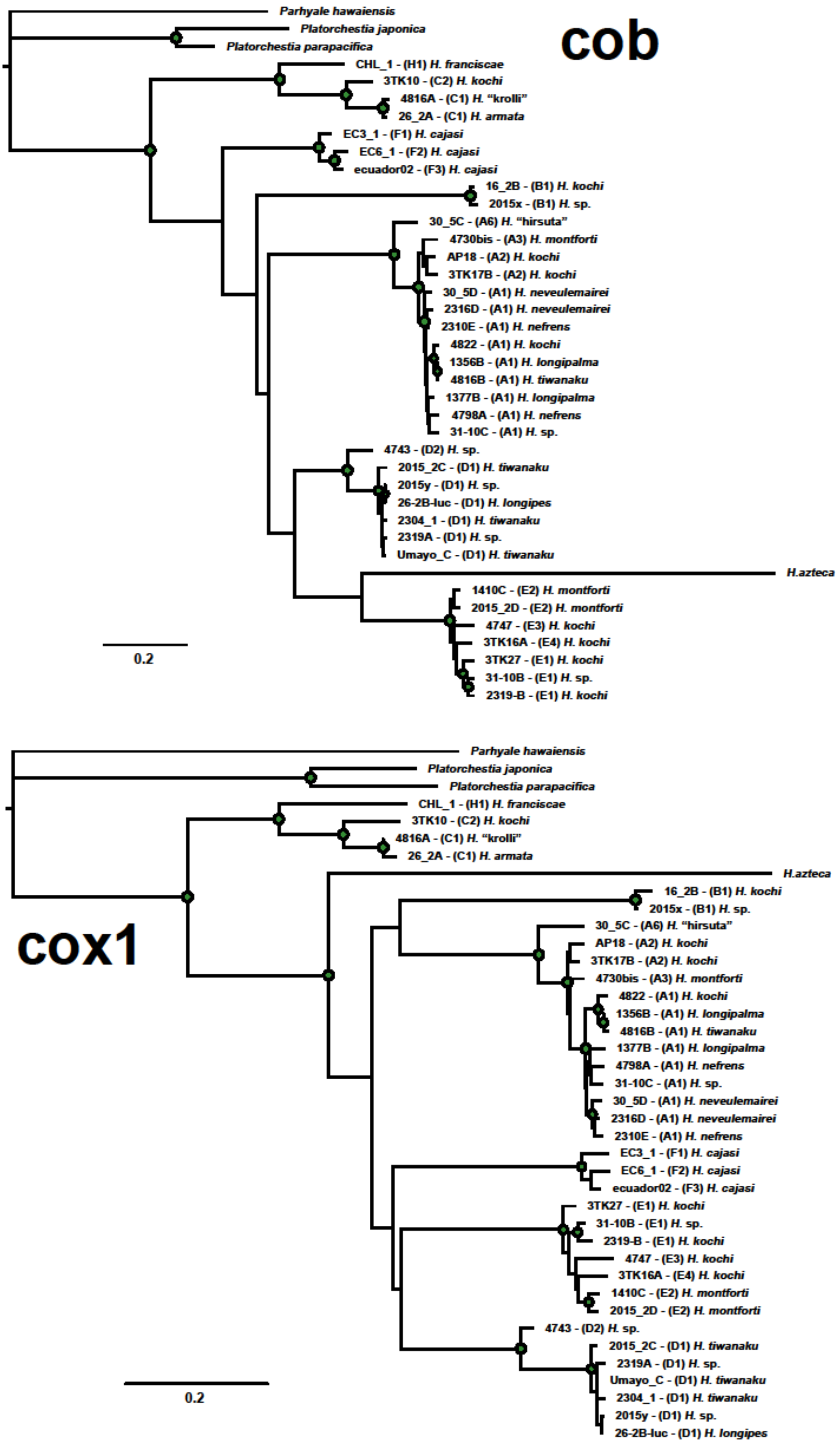
cob
Parhyale hawaiensis
Platorchestia japonica
Platorchestia parapacifica
CHL_1 - (H1) H. franciscae
3TK10 - (C2) H. kochi
4816A - (C1) H. "krolli"
26_2A - (C1) H. armata
EC3_1 - (F1) H. cajasi
EC6_1 - (F2) H. cajasi
ecuador02 - (F3) H. cajasi
16_2B - (B1) H. kochi
2015x - (B1) H. sp.
30_5C - (A6) H. "hirsuta"
4730bis - (A3) H. montforti
AP18 - (A2) H. kochi
3TK17B - (A2) H. kochi
30_5D - (A1) H. neveulemairei
2316D - (A1) H. neveulemairei
2310E - (A1) H. nefrens
4822 - (A1) H. kochi
1356B - (A1) H. longipalma
4816B - (A1) H. tiwanaku
1377B - (A1) H. longipalma
4798A - (A1) H. nefrens
31-10C - (A1) H. sp.
4743 - (D2) H. sp.
2015_2C - (D1) H. tiwanaku
2015y - (D1) H. sp.
26-2B-luc - (D1) H. longipes
2304_1 - (D1) H. tiwanaku
2319A - (D1) H. sp.
Umayo_C - (D1) H. tiwanaku
H.azteca
1410C - (E2) H. montforti
2015_2D - (E2) H. montforti
4747 - (E3) H. kochi
3TK16A - (E4) H. kochi
3TK27 - (E1) H. kochi
31-10B - (E1) H. sp.
2319-B - (E1) H. kochi
0.2
cox1
Parhyale hawaiensis
Platorchestia japonica
Platorchestia parapacifica
CHL_1 - (H1) H. franciscae
3TK10 - (C2) H. kochi
4816A - (C1) H. "krolli"
26_2A - (C1) H. armata
H.azteca
16_2B - (B1) H. kochi
2015x - (B1) H. sp.
30_5C - (A6) H. "hirsuta"
AP18 - (A2) H. kochi
3TK17B - (A2) H. kochi
4730bis - (A3) H. montforti
4822 - (A1) H. kochi
1356B - (A1) H. longipalma
4816B - (A1) H. tiwanaku
1377B - (A1) H. longipalma
4798A - (A1) H. nefrens
31-10C - (A1) H. sp.
30_5D - (A1) H. neveulemairei
2316D - (A1) H. neveulemairei
2310E - (A1) H. nefrens
EC3_1 - (F1) H. cajasi
EC6_1 - (F2) H. cajasi
ecuador02 - (F3) H. cajasi
3TK27 - (E1) H. kochi
31-10B - (E1) H. sp.
2319-B - (E1) H. kochi
4747 - (E3) H. kochi
3TK16A - (E4) H. kochi
1410C - (E2) H. montforti
2015_2D - (E2) H. montforti
4743 - (D2) H. sp.
2015_2C - (D1) H. tiwanaku
2319A - (D1) H. sp.
Umayo_C - (D1) H. tiwanaku
2304_1 - (D1) H. tiwanaku
2015y - (D1) H. sp.
26-2B-luc - (D1) H. longipes
0.2

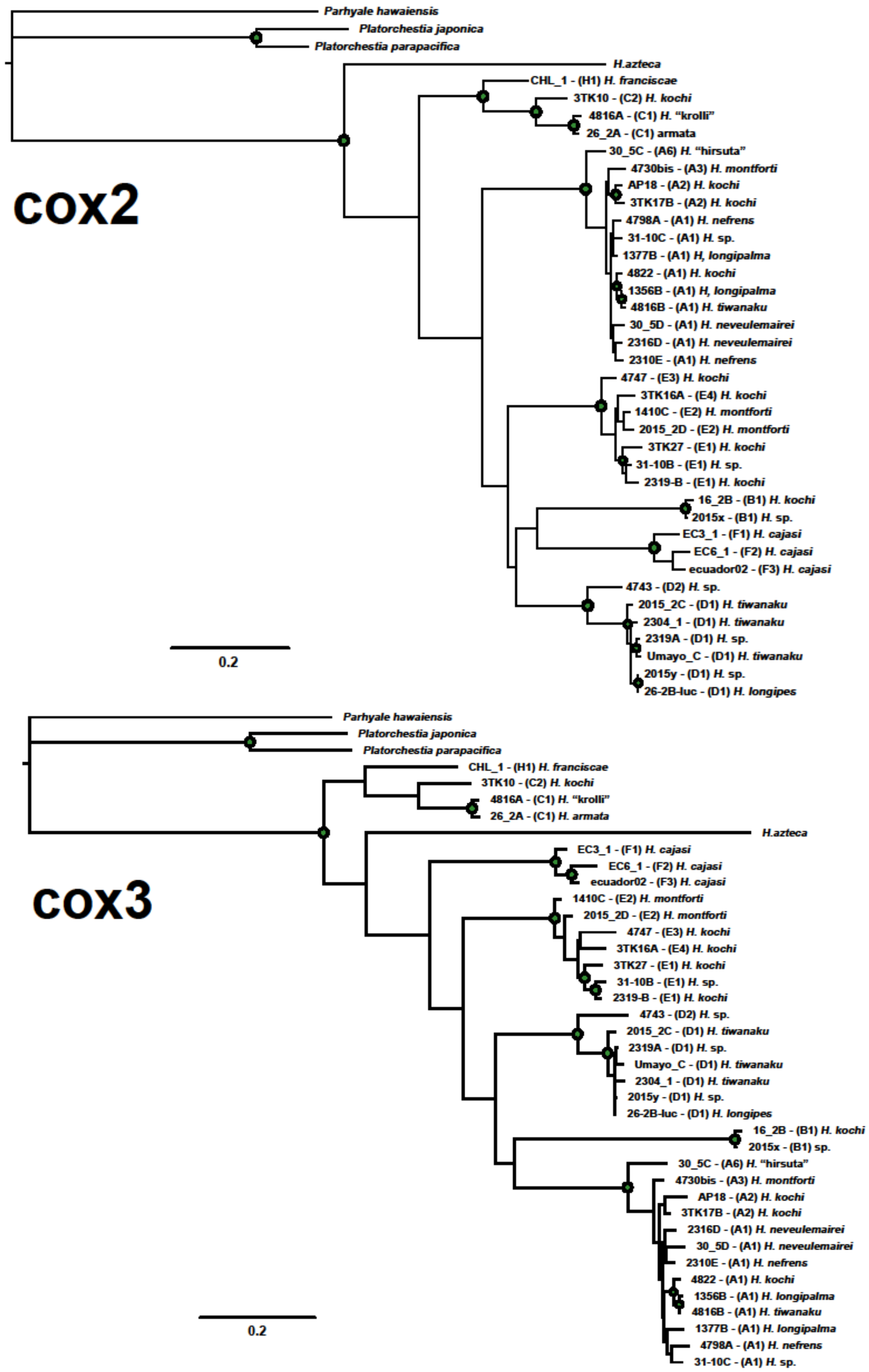
cox2
Parhyale hawaiensis
Platorchestia japonica
Platorchestia parapacifica
H.azteca
CHL_1 - (H1) H. franciscae
3TK10 - (C2) H. kochi
4816A - (C1) H. "krolli"
26_2A - (C1) armata
30_5C - (A6) H. "hirsuta"
4730bis - (A3) H. montforti
AP18 - (A2) H. kochi
3TK17B - (A2) H. kochi
4798A - (A1) H. nefrens
31-10C - (A1) H. sp.
1377B - (A1) H, longipalma
4822 - (A1) H. kochi
1356B - (A1) H, longipalma
4816B - (A1) H. tiwanaku
30_5D - (A1) H. neveulemairei
2316D - (A1) H. neveulemairei
2310E - (A1) H. nefrens
4747 - (E3) H. kochi
3TK16A - (E4) H. kochi
1410C - (E2) H. montforti
2015_2D - (E2) H. montforti
3TK27 - (E1) H. kochi
31-10B - (E1) H. sp.
2319-B - (E1) H. kochi
16_2B - (B1) H. kochi
2015x - (B1) H. sp.
EC3_1 - (F1) H. cajasi
EC6_1 - (F2) H. cajasi
ecuador02 - (F3) H. cajasi
4743 - (D2) H. sp.
2015_2C - (D1) H. tiwanaku
2304_1 - (D1) H. tiwanaku
2319A - (D1) H. sp.
Umayo_C - (D1) H. tiwanaku
2015y - (D1) H. sp.
26-2B-luc - (D1) H. longipes
0.2
cox3
Parhyale hawaiensis
Platorchestia japonica
Platorchestia parapacifica
CHL_1 - (H1) H. franciscae
3TK10 - (C2) H. kochi
4816A - (C1) H. "krolli"
26_2A - (C1) H. armata
H.azteca
EC3_1 - (F1) H. cajasi
EC6_1 - (F2) H. cajasi
ecuador02 - (F3) H. cajasi
1410C - (E2) H. montforti
2015_2D - (E2) H. montforti
4747 - (E3) H. kochi
3TK16A - (E4) H. kochi
3TK27 - (E1) H. kochi
31-10B - (E1) H. sp.
2319-B - (E1) H. kochi
4743 - (D2) H. sp.
2015_2C - (D1) H. tiwanaku
2319A - (D1) H. sp.
Umayo_C - (D1) H. tiwanaku
2304_1 - (D1) H. tiwanaku
2015y - (D1) H. sp.
26-2B-luc - (D1) H. longipes
16_2B - (B1) H. kochi
2015x - (B1) sp.
30_5C - (A6) H. "hirsuta"
4730bis - (A3) H. montforti
AP18 - (A2) H. kochi
3TK17B - (A2) H. kochi
2316D - (A1) H. neveulemairei
30_5D - (A1) H. neveulemairei
2310E - (A1) H. nefrens
4822 - (A1) H. kochi
1356B - (A1) H. longipalma
4816B - (A1) H. tiwanaku
1377B - (A1) H. longipalma
4798A - (A1) H. nefrens
31-10C - (A1) H. sp.
0.2

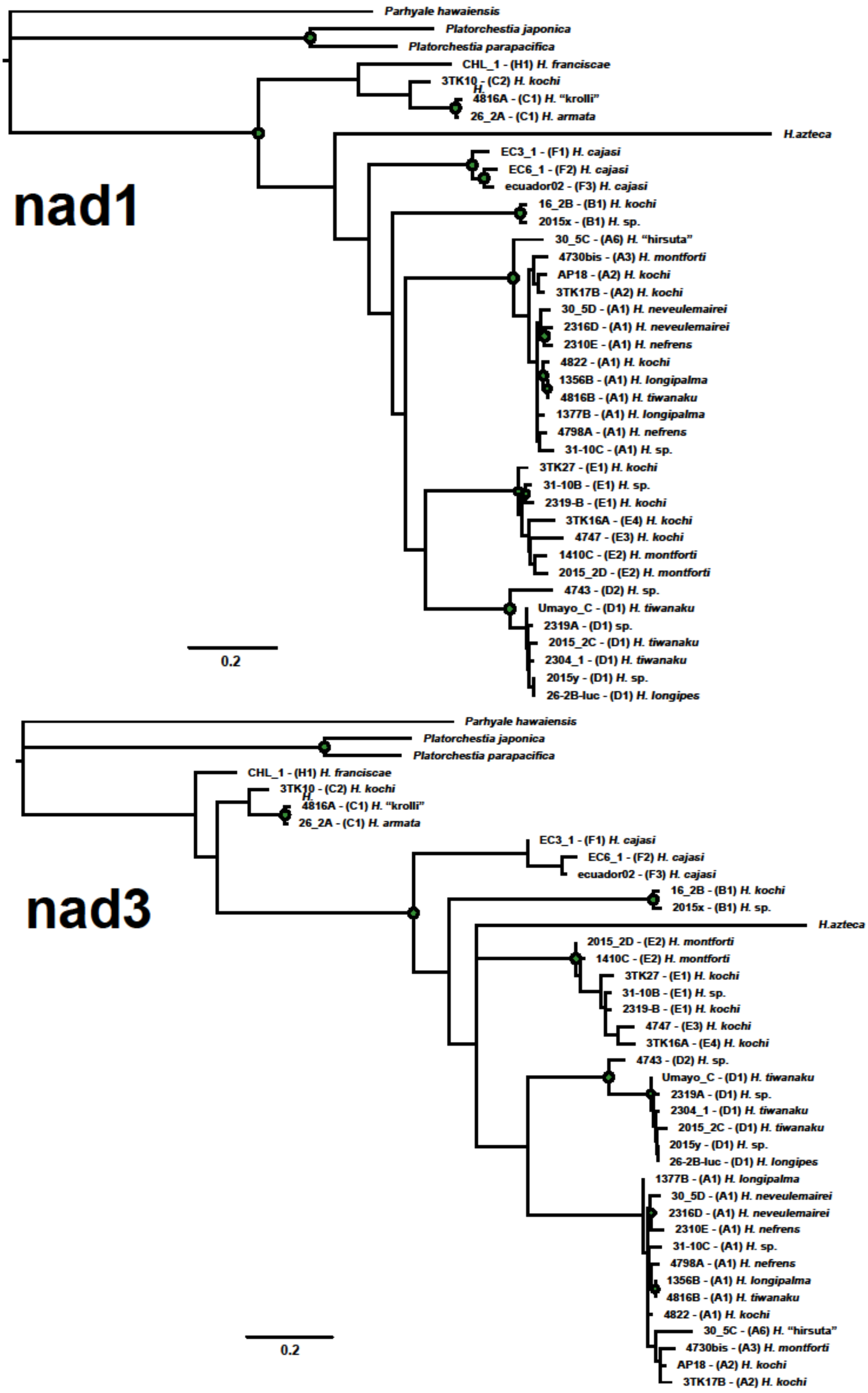
nad1
Parhyale hawaiensis
Platorchestia japonica
Platorchestia parapacifica
CHL_1 - (H1) H. franciscae
3TK10 - (C2) H. kochi
4816A - (C1) H. "krolli"
26_2A - (C1) H. armata
H.azteca
EC3_1 - (F1) H. cajasi
EC6_1 - (F2) H. cajasi
ecuador02 - (F3) H. cajasi
16_2B - (B1) H. kochi
2015x - (B1) H. sp.
30_5C - (A6) H. "hirsuta"
4730bis - (A3) H. montforti
AP18 - (A2) H. kochi
3TK17B - (A2) H. kochi
30_5D - (A1) H. neveulemairei
2316D - (A1) H. neveulemairei
2310E - (A1) H. nefrens
4822 - (A1) H. kochi
1356B - (A1) H. longipalma
4816B - (A1) H. tiwanaku
1377B - (A1) H. longipalma
4798A - (A1) H. nefrens
31-10C - (A1) H. sp.
3TK27 - (E1) H. kochi
31-10B - (E1) H. sp.
2319-B - (E1) H. kochi
3TK16A - (E4) H. kochi
4747 - (E3) H. kochi
1410C - (E2) H. montforti
2015_2D - (E2) H. montforti
4743 - (D2) H. sp.
Umayo_C - (D1) H. tiwanaku
2319A - (D1) sp.
2015_2C - (D1) H. tiwanaku
2304_1 - (D1) H. tiwanaku
2015y - (D1) H. sp.
26-2B-luc - (D1) H. longipes
0.2
nad3
Parhyale hawaiensis
Platorchestia japonica
Platorchestia parapacifica
CHL_1 - (H1) H. franciscae
3TK10 - (C2) H. kochi
4816A - (C1) H. "krolli"
26_2A - (C1) H. armata
EC3_1 - (F1) H. cajasi
EC6_1 - (F2) H. cajasi
ecuador02 - (F3) H. cajasi
16_2B - (B1) H. kochi
2015x - (B1) H. sp.
H.azteca
2015_2D - (E2) H. montforti
1410C - (E2) H. montforti
3TK27 - (E1) H. kochi
31-10B - (E1) H. sp.
2319-B - (E1) H. kochi
4747 - (E3) H. kochi
3TK16A - (E4) H. kochi
4743 - (D2) H. sp.
Umayo_C - (D1) H. tiwanaku
2319A - (D1) H. sp.
2304_1 - (D1) H. tiwanaku
2015_2C - (D1) H. tiwanaku
2015y - (D1) H. sp.
26-2B-luc - (D1) H. longipes
1377B - (A1) H. longipalma
30_5D - (A1) H. neveulemairei
2316D - (A1) H. neveulemairei
2310E - (A1) H. nefrens
31-10C - (A1) H. sp.
4798A - (A1) H. nefrens
1356B - (A1) H. longipalma
4816B - (A1) H. tiwanaku
4822 - (A1) H. kochi
30_5C - (A6) H. "hirsuta"
4730bis - (A3) H. montforti
AP18 - (A2) H. kochi
3TK17B - (A2) H. kochi
0.2

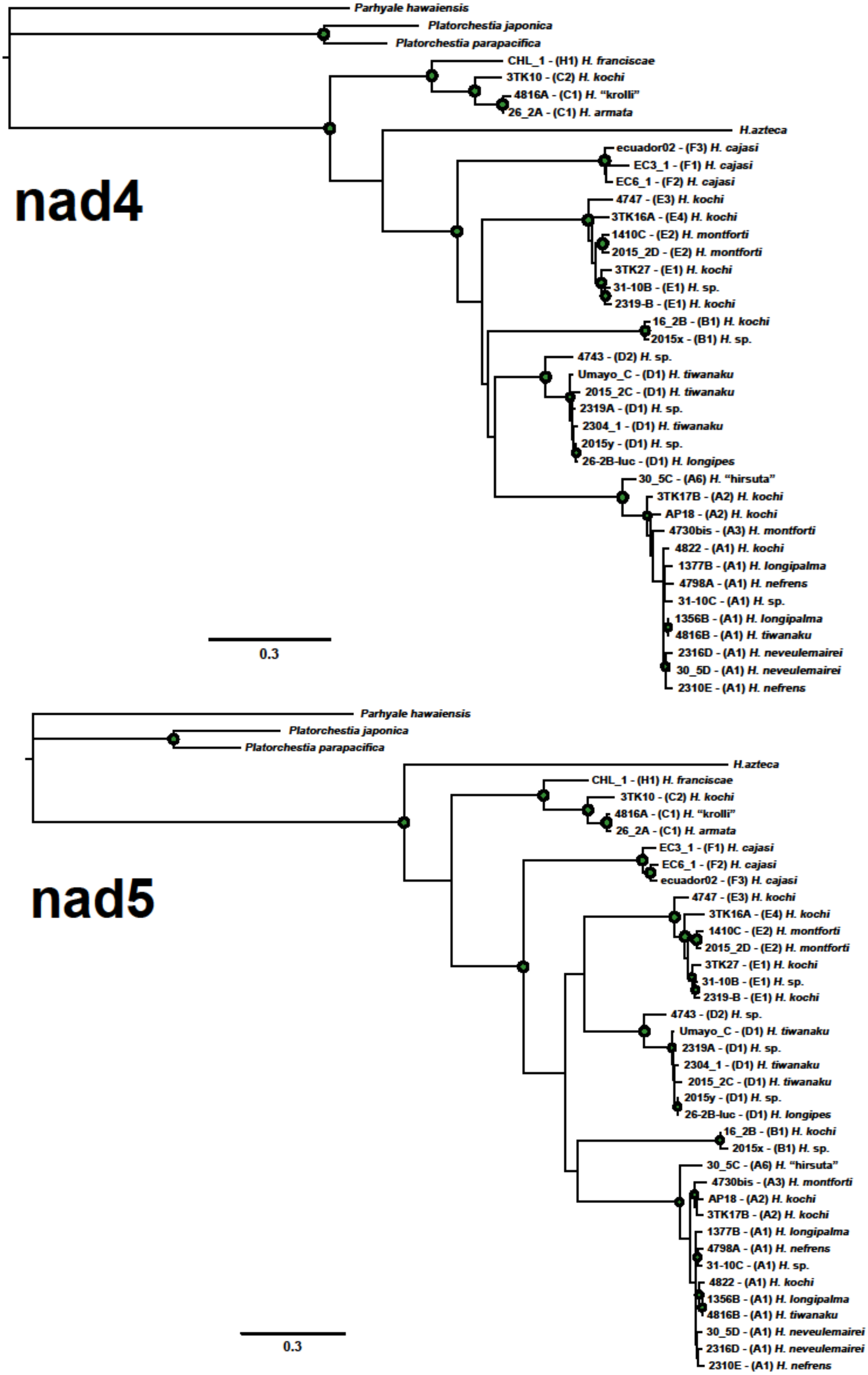
Parhyale hawaiensis
Platorchestia japonica
Platorchestia parapacifica
CHL_1 - (H1) H. franciscae
3TK10 - (C2) H. kochi
4816A - (C1) H. "krolli"
26_2A - (C1) H. armata
H.azteca
ecuador02 - (F3) H. cajasi
EC3_1 - (F1) H. cajasi
EC6_1 - (F2) H. cajasi
nad4
4747 - (E3) H. kochi
3TK16A - (E4) H. kochi
1410C - (E2) H. montforti
2015_2D - (E2) H. montforti
3TK27 - (E1) H. kochi
31-10B - (E1) H. sp.
2319-B - (E1) H. kochi
16_2B - (B1) H. kochi
2015x - (B1) H. sp.
4743 - (D2) H. sp.
Umayo_C - (D1) H. tiwanaku
2015_2C - (D1) H. tiwanaku
2319A - (D1) H. sp.
2304_1 - (D1) H. tiwanaku
2015y - (D1) H. sp.
26-2B-luc - (D1) H. longipes
30_5C - (A6) H. "hirsuta"
3TK17B - (A2) H. kochi
AP18 - (A2) H. kochi
4730bis - (A3) H. montforti
4822 - (A1) H. kochi
1377B - (A1) H. longipalma
4798A - (A1) H. nefrens
31-10C - (A1) H. sp.
1356B - (A1) H. longipalma
4816B - (A1) H. tiwanaku
2316D - (A1) H. neveulemairei
30_5D - (A1) H. neveulemairei
2310E - (A1) H. nefrens
0.3
Parhyale hawaiensis
Platorchestia japonica
Platorchestia parapacifica
H.azteca
CHL_1 - (H1) H. franciscae
3TK10 - (C2) H. kochi
4816A - (C1) H. "krolli"
26_2A - (C1) H. armata
EC3_1 - (F1) H. cajasi
EC6_1 - (F2) H. cajasi
ecuador02 - (F3) H. cajasi
nad5
4747 - (E3) H. kochi
3TK16A - (E4) H. kochi
1410C - (E2) H. montforti
2015_2D - (E2) H. montforti
3TK27 - (E1) H. kochi
31-10B - (E1) H. sp.
2319-B - (E1) H. kochi
4743 - (D2) H. sp.
Umayo_C - (D1) H. tiwanaku
2319A - (D1) H. sp.
2304_1 - (D1) H. tiwanaku
2015_2C - (D1) H. tiwanaku
2015y - (D1) H. sp.
26-2B-luc - (D1) H. longipes
16_2B - (B1) H. kochi
2015x - (B1) H. sp.
30_5C - (A6) H. "hirsuta"
4730bis - (A3) H. montforti
AP18 - (A2) H. kochi
3TK17B - (A2) H. kochi
1377B - (A1) H. longipalma
4798A - (A1) H. nefrens
31-10C - (A1) H. sp.
4822 - (A1) H. kochi
1356B - (A1) H. longipalma
4816B - (A1) H. tiwanaku
30_5D - (A1) H. neveulemairei
2316D - (A1) H. neveulemairei
2310E - (A1) H. nefrens
0.3

Supplementary Figure 3. Tanglegram of the mitochondrial and single-copy nuclear trees obtained with the dendextend package in R (Galil 2015). The BEAST tree topology, based on the 13 mitochondrial concatenated PCGs and using the single-copy nuclear gene-fragments concatenate, are shown on the left and right, respectively. Branches in black, green or red indicate subtrees present in both tree topologies. Dashed lines highlight nodes not present in the alternative tree topology.

Galili, T. Dendextend: An R package for visualizing, adjusting and comparing trees of hierarchical clustering. *Bioinformatics* **31**, 3718–3720 (2015).

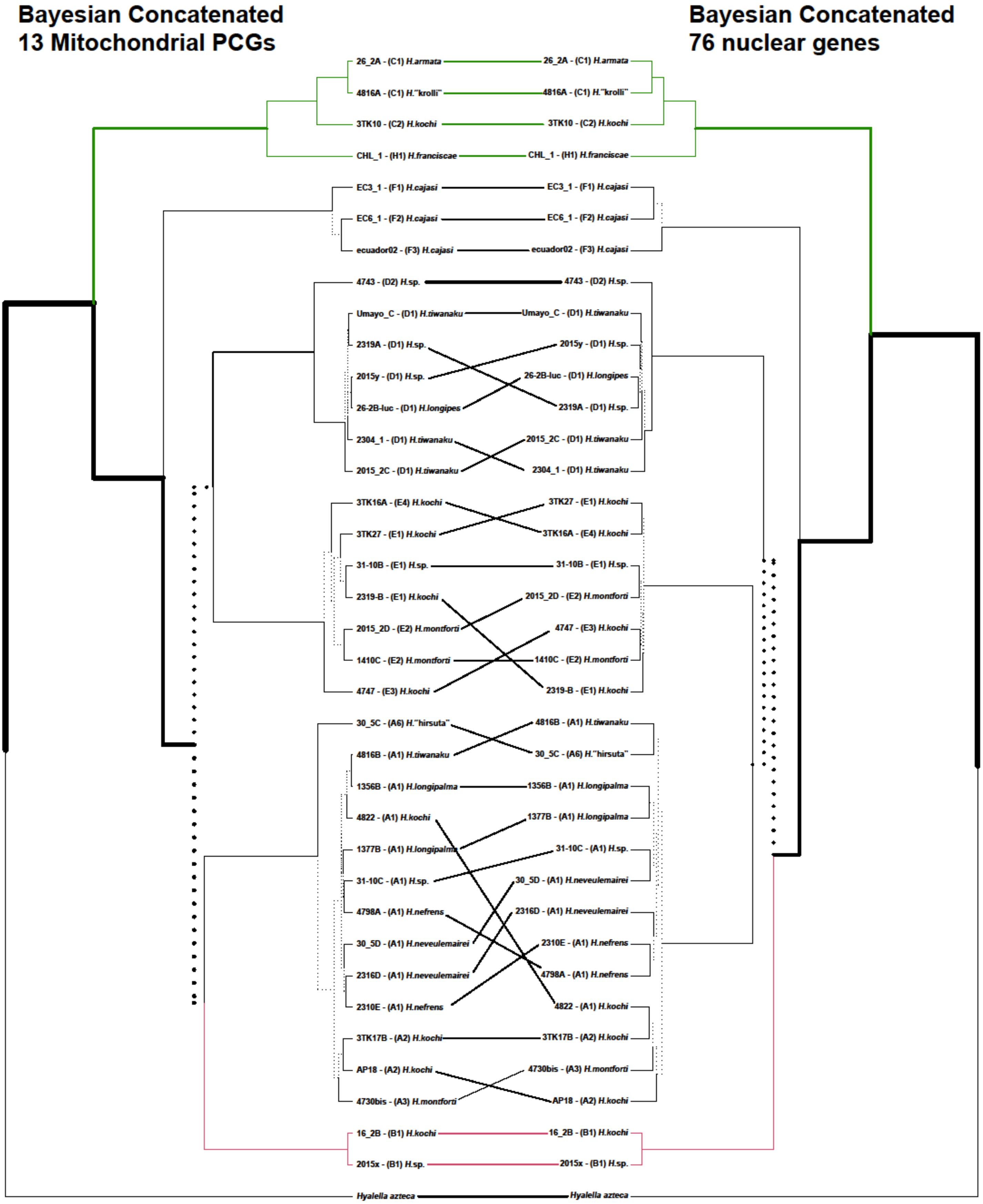

Bayesian Concatenated
13 Mitochondrial PCGs
Bayesian Concatenated
76 nuclear genes
26_2A - (C1) H.armata
4816A - (C1) H."krolli"
3TK10 - (C2) H.kochi
CHL_1 - (H1) H.franciscae
EC3_1 - (F1) H.cajasi
EC6_1 - (F2) H.cajasi
ecuador02 - (F3) H.cajasi
4743 - (D2) H.sp.
Umayo_C - (D1) H.tiwanaku
2319A - (D1) H.sp.
2015y - (D1) H.sp.
26-2B-luc - (D1) H.longipes
2304_1 - (D1) H.tiwanaku
2015_2C - (D1) H.tiwanaku
3TK16A - (E4) H.kochi
3TK27 - (E1) H.kochi
31-10B - (E1) H.sp.
2319-B - (E1) H.kochi
2015_2D - (E2) H.montforti
1410C - (E2) H.montforti
4747 - (E3) H.kochi
30_5C - (A6) H."hirsuta"
4816B - (A1) H.tiwanaku
1356B - (A1) H.longipalma
4822 - (A1) H.kochi
1377B - (A1) H.longipalma
31-10C - (A1) H.sp.
4798A - (A1) H.nefrens
30_5D - (A1) H.neveulemairei
2316D - (A1) H.neveulemairei
2310E - (A1) H.nefrens
3TK17B - (A2) H.kochi
AP18 - (A2) H.kochi
4730bis - (A3) H.montforti
16_2B - (B1) H.kochi
2015x - (B1) H.sp.
Hyalella azteca
26_2A - (C1) H.armata
4816A - (C1) H."krolli"
3TK10 - (C2) H.kochi
CHL_1 - (H1) H.franciscae
EC3_1 - (F1) H.cajasi
EC6_1 - (F2) H.cajasi
ecuador02 - (F3) H.cajasi
4743 - (D2) H.sp.
Umayo_C - (D1) H.tiwanaku
2015y - (D1) H.sp.
26-2B-luc - (D1) H.longipes
2319A - (D1) H.sp.
2015_2C - (D1) H.tiwanaku
2304_1 - (D1) H.tiwanaku
3TK27 - (E1) H.kochi
3TK16A - (E4) H.kochi
31-10B - (E1) H.sp.
2015_2D - (E2) H.montforti
4747 - (E3) H.kochi
1410C - (E2) H.montforti
2319-B - (E1) H.kochi
4816B - (A1) H.tiwanaku
30_5C - (A6) H."hirsuta"
1356B - (A1) H.longipalma
1377B - (A1) H.longipalma
31-10C - (A1) H.sp.
30_5D - (A1) H.neveulemairei
2316D - (A1) H.neveulemairei
2310E - (A1) H.nefrens
4798A - (A1) H.nefrens
4822 - (A1) H.kochi
3TK17B - (A2) H.kochi
4730bis - (A3) H.montforti
AP18 - (A2) H.kochi
16_2B - (B1) H.kochi
2015x - (B1) H.sp.
Hyalella azteca

Supplementary Figure 4. Phylogenetic tree based on the single-copy nuclear genes obtained with ASTRAL III. The terminals were classified into species following Jurado-Rivera et al. (2020) with letters and numbers in tips corresponding to Molecular Taxonomic Operational Units. Circles indicate nodes with maximum nodal support.

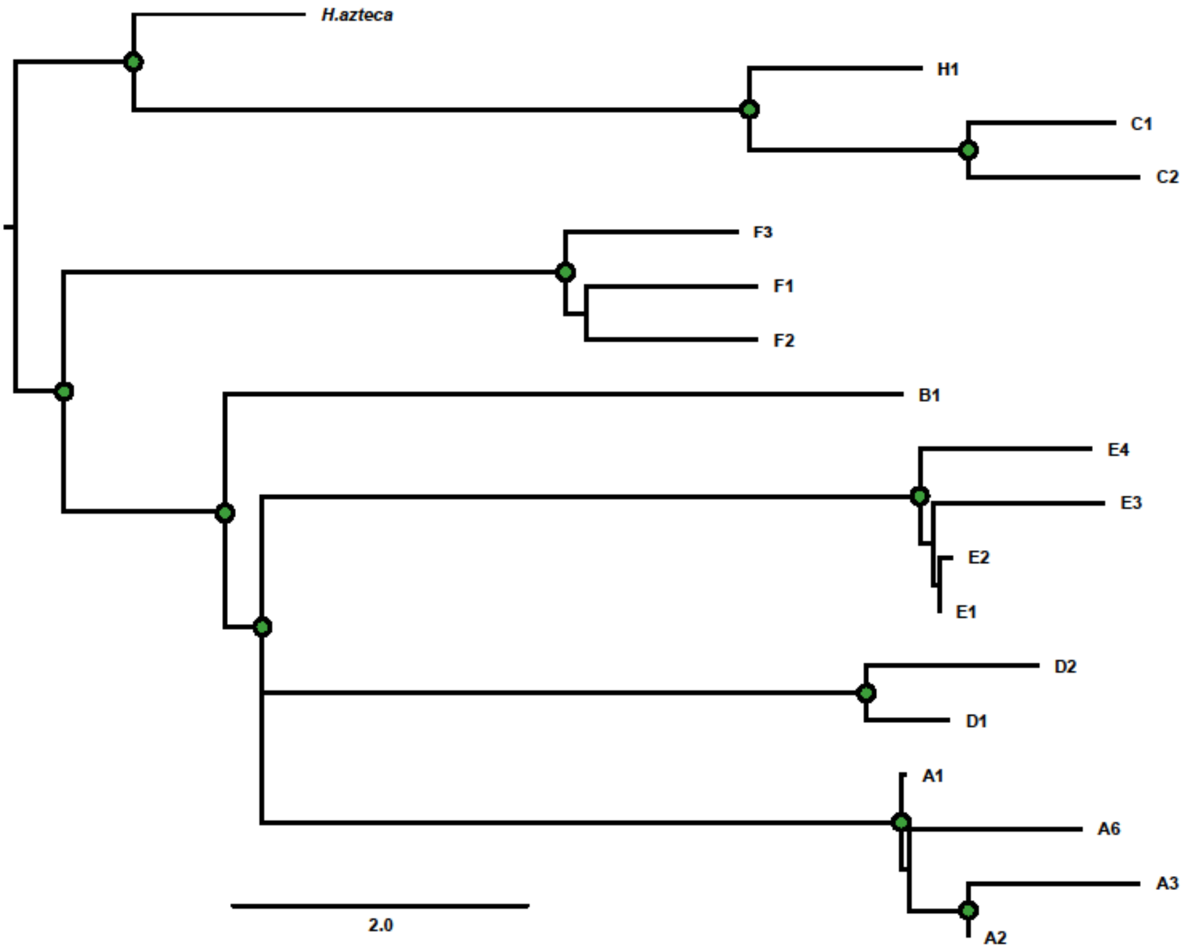

Tab. 1

Sample	complete	AT%	nad2 (+)	cox1 (+)	cox2 (+)	atp8 (+)	atp6 (+)	cox3 (+)	nad3 (+)	nad5 (-)	nad4 (-)	nad4L (-)	nad6 (+)	cob (+)	nad1 (-)
**1356B**	15.177	67.9%	982	1539	681	156	672	789	354	1714	1312	294	486	1134	936
**1377B**	15.077	67.9%	982	1539	681	156	672	789	354	1714	1312	294	486	1134	936
**1410C***	14.277		982	1539	681	156	672	789	354	1717	1312	294	486	1134	936
**2015_2C**	14.956	70.2%	982	1539	681	156	672	789	354	1714	1312	294	486	1134	936
**2015_2D***	14.310		982	1539	681	156	672	789	354	1717	1312	294	486	1134	936
**2015x***	9.904		982	1539	681	156	672	789	354	1714	1312	294	**255****	1134	936
**2015y**	14.965	70.5%	982	1539	681	156	672	789	354	1714	1312	294	486	1134	936
**2304**	14.963	70.5%	982	1539	681	156	672	789	354	1714	1312	294	486	1134	936
**2310_E**	15.101	73.9%	982	1539	681	156	672	789	354	1714	1312	294	486	1134	936
**2316D***	5.763		982	1539	681	156	672	789	354	1714	1312	294	486	1134	936
**2319A**	14.953	70.5%	982	1539	681	156	672	789	354	1714	1312	294	486	1134	936
**2319B***	15.016		982	1539	681	156	672	789	354	1717	1312	294	486	1134	936
**16_2B***	5.632		982	1539	681	156	672	789	354	1714	1312	294	486	1134	936
**26_2A***	14.069		982	1539	681	156	672	789	354	1714	1312	294	486	1134	936
**30_5C**	15.204	67.9%	982	1539	681	156	672	789	354	1714	1312	294	486	1134	936
**30_5D***	13.991		982	1539	681	156	672	789	354	1714	1312	294	486	1134	936
**31_10B***	14.474		982	1539	681	156	672	789	354	1717	1312	294	486	1134	936
**31_10C***	7.253		982	1539	681	156	672	789	354	**1626****	1312	294	**381****	1134	936
**3TK10***	13.968		982	1539	681	156	672	789	354	1714	1312	294	486	1134	936
**3TK16A***	14.182		982	1539	681	156	672	789	354	1717	1312	294	486	1134	936
**3TK17B***	14.723		982	1539	681	156	672	789	354	1714	1312	294	486	1134	936
**3TK27***	14.175		982	1539	681	156	672	789	354	1717	1312	294	486	1134	936
**4730bis**	14.937	68.1%	982	1539	681	156	672	789	354	1714	1312	294	486	1134	936
**4743***	13.991		982	1539	681	156	672	789	354	1714	1312	294	486	1134	936
**4747***	14.448		982	1539	681	156	672	789	354	1717	1312	294	486	1134	936
**4798A**	14.999	67.9%	982	1539	681	156	672	789	354	1714	1312	294	486	1134	936
**4816A***	7.656		982	1539	681	156	672	789	354	**1528****	**816****	294	486	1134	936
**4816B**	14.942	67.9%	982	1539	681	156	672	789	354	1714	1312	294	486	1134	936
**4822***	14.152		982	1539	681	156	672	789	354	1714	1312	294	486	1134	936
**AP-18***	14.056		982	1539	681	156	672	789	354	1714	1312	294	486	1134	936
**CHL-1***	14.459		982	1539	681	156	672	789	354	1714	1312	294	486	1134	936
**EC6-1**	14.914	66.9%	982	1539	681	156	672	789	354	1714	1312	294	486	1134	936
**EC3-1**	14.930	67.1%	982	1539	681	156	672	789	354	1714	1312	294	486	1134	936
**ecuador02***	13.972		982	1539	681	156	672	789	354	1714	1312	294	486	1134	936
**Umayo-C**	15.037	70,6%	982	1539	681	156	672	789	354	1714	1312	294	486	1134	936

Tab. 1 (cont.)

Sample	C (+)	Y (-)	M (+)	W (+)	G (+)	L2 (+)	K (+)	D (+)	S1 (+)	E (+)	F (-)	H (-)
1356B	57	64	62	63	63	61	62	62	54	62	61	62
1377B	57	64	62	63	63	61	62	62	54	62	61	62
1410C*	-	-	63	63	63	61	62	61	52	63	61	62
2015_2C	57	59	62	63	63	61	62	62	51	62	61	62
2015_2D*	57	-	63	63	63	61	62	60	52	63	61	62
2015x*	-	-	-	63	63	61	-	-	-	-	61	62
2015y	57	59	62	63	63	61	62	62	51	62	61	62
2304	57	59	62	63	63	61	62	62	51	62	61	62
2310_E	57	65	62	63	63	61	62	62	54	63	61	62
2316D*	-	-	62	63	63	61	62	62	54	62	-	-
2319A	57	59	62	63	63	61	62	62	51	62	61	62
2319B*	-	58	63	63	63	61	62	62	51	63	61	62
16_2B*	-	-	62	-	-	61	62	62	52	61	-	-
26_2A*	57	-	-	63	63	61	63	61	51	65	61	62
30_5C	57	59	62	63	63	61	62	62	54	62	61	62
30_5D*	-	-	62	63	63	61	62	60	54	62	61	62
31_10B*	57	59	63	63	63	61	62	60	52	63	61	62
31_10C*	-	-	-	63	63	61	62	61	54	62	-	-
3TK10*	-	-	-	63	63	61	63	61	52	65	61	62
3TK16A*	-	-	63	63	63	61	62	60	52	63	61	62
3TK17B*	-	66	62	63	63	61	62	60	52	62	61	62
3TK27*	-	-	63	63	63	61	62	60	52	63	61	62
4730bis	57	64	62	63	63	61	62	62	54	62	61	62
4743*	-	-	62	63	63	61	62	60	51	62	61	62
4747*	-	-	63	63	63	61	62	60	51	63	61	62
4798A	57	64	62	63	63	61	62	62	54	63	61	62
4816A*	-	-	-	63	63	61	62	61	51	65	61	-
4816B	57	64	62	63	63	61	62	62	54	62	61	62
4822*	-	-	62	63	63	61	62	60	54	62	61	62
AP-18*	-	-	62	63	63	61	62	61	54	62	61	62
CHL-1*	57	61	62	63	63	61	63	60	51	65	61	62
EC6-1	57	59	63	63	63	61	62	62	51	63	61	65
EC3-1	57	59	62	63	63	61	62	62	51	63	61	65
ecuador02*	-	-	63	63	63	61	62	60	54	63	61	65
Umayo-C	57	59	62	63	63	61	62	62	51	62	61	62

Sample	P (-)	I (+)	Q (-)	A (+)	N (+)	R (+)	T (+)	S2 (+)	L1 (-)	V (-)	16S (-)	12S (-)
**1356B**	61	60	60	60	63	57	60	60	60	51	995	645
**1377B**	61	60	60	60	63	57	60	60	60	51	995	645
**1410C***	62	59	60	60	64	57	60	62	60	51	999	645
**2015_2C**	62	61	60	60	63	57	60	60	60	51	1003	646
**2015_2D***	62	59	60	60	64	57	60	62	60	51	999	645
**2015x***	61	61	-	-	-	-	-	-	-	-	-	-
**2015y**	62	61	60	60	63	56	60	60	60	51	998	645
**2304**	62	61	60	60	63	56	60	60	60	51	998	645
**2310_E**	61	60	60	60	63	57	60	60	60	51	995	645
**2316D***	-	-	-	-	-	-	-	-	-	-	-	-
**2319A**	62	61	60	60	63	56	60	60	60	51	998	644
**2319B***	62	59	60	60	64	57	60	62	60	51	999	645
**16_2B***	-	-	-	-	-	-	-	-	-	-	-	-
**26_2A***	61	61	60	60	63	57	60	61	60	51	1000	647
**30_5C**	61	61	60	60	63	57	60	60	60	51	996	645
**30_5D***	61	60	60	60	63	57	60	60	60	51	995	645
**31_10B***	62	59	60	60	64	57	60	62	60	51	999	645
**31_10C***	-	-	-	-	-	-	-	-	-	-	-	-
**3TK10***	61	61	60	60	63	57	60	61	60	51	1000	648
**3TK16A***	62	59	60	60	64	57	60	62	60	51	999	645
**3TK17B***	61	60	60	60	63	57	60	60	60	51	996	645
**3TK27***	62	59	60	60	64	57	60	62	60	51	999	645
**4730bis**	61	60	60	60	63	57	60	61	60	51	995	645
**4743***	62	61	60	60	63	56	60	60	60	51	998	648
**4747***	62	59	60	60	64	57	60	62	60	51	999	644
**4798A**	61	60	60	60	63	57	60	60	60	51	995	645
**4816A***	-	-	-	-	-	-	-	-	-	-	-	-
**4816B**	61	60	60	60	63	57	60	60	60	51	995	645
**4822***	61	60	60	60	63	57	60	60	60	51	995	644
**AP-18***	61	60	60	60	63	57	60	60	60	51	995	646
**CHL-1***	61	61	60	60	63	57	60	61	60	51	998	646
**EC6-1**	62	61	59	60	63	56	60	61	60	51	1001	646
**EC3-1**	62	61	59	60	63	56	60	61	60	51	1001	646
**ecuador02***	62	61	59	60	63	56	60	61	60	51	1000	646
**Umayo-C**	62	61	60	60	63	56	60	60	60	51	998	645

Supplementary Table 1. Mitochondrial genome sequence length, AT% (only for the complete ones) and missing data for the species included in the analyses. The - , * and ** symbols indicate cases of missing features, incomplete mitogenomes and genes, respectively.

No Outgroups			
Tree	logL	deltaL	p-AU
1	-106549	0.026549	0.597
2	-106549	0	0.59
3	-106555	6.531	0.235
4	-106549	0.60502	0.676
5	-106549	0.26046	0.599
6	-106555	61.546	0.172
7	-106549	0.079716	0.623
With Outgroups			
Tree	logL	deltaL	p-AU
1	-128157	0.024419	0.634
2	-128157	0	0.637
3	-128164	69.543	0.242
4	-128157	0.43518	0.677
5	-128157	0.04285	0.683
6	-128163	63.273	0.2
7	-128188	31.275	0.0168
with Platorchestia as outgrup only (i.e. Parhyale removed)			
Tree	logL	deltaL	p-AU
1	-120459	0.22953	0.642
2	-120459	0.21276	0.647
3	-120467	82.344	0.185
4	-120459	0.73091	0.637
5	-120459	0	0.724
6	-120465	61.364	0.195
7	-120481	22.67	0.0294

Supplementary Table 2. Approximately Unbiased tests (AU-tests) contrasting alternative ML topologies of the 13 concatenated mitochondrial PCGs with the Bayesian topology using the codons model with codons equal (Supplemantary Fig. 2c). The "No outgroups" tree excluded *Parhyale hawaiensis* and the two *Platorchestia* species from the analysis. "With outgroups" used all the outgroup species. The last tree used *Platorchestia* only. Blue cells indicate that the null hypothesis H_0 (the two topologies are not significantly different) is rejected. The seven topologies tested are from alternative trees showed previously (see Supplementary Fig. 1). Tree 1: Maximum Likelihood tree inferred with IQ-TREE implementing partitions by codon position (see Figure 2a of main text). Tree 2: single partition tree obtained with IQ-TREE (see Supplementary Figure 1a). Tree 3: Bayesian tree obtained with MrBayes implementing the codons model with codons equal (see Supplementary Figure 1c). Tree 4: Bayesian tree inferred with MrBayes implementing the codons model with model GY94 (see Supplementary Figure 1d). Tree 5: Bayesian tree inferred with MrBayes implementing the codons model with model M3 (see Supplementary Figure 1e). Tree 6: Maximum Likelihood amino-acid tree obtained with MrBayes with model mt-MET+I+F+G4 (see Supplementary Figure 1f). Tree 7: the Maximum Likelihood tree implementing partitions by codon position in which *H. azteca* has been forced as a sister group of the South-American *Hyalella*.

Tab. 3

Gene Code	Highest match	Submission code	max score	E value	Perc. ID
OG0039918	-	-	-	-	-
OG0039932	PREDICTED: Hyalella azteca uncharacterized LOC108668661 (LOC108668661), mRNA	XM_018155904.1	348	2E-91	82,37%
OG0040004	PREDICTED: Hyalella azteca uncharacterized LOC108678788 (LOC108678788), mRNA	XM_018167255.1	594	1E-165	85,69%
OG0040013	PREDICTED: Hyalella azteca uncharacterized LOC108671168 (LOC108671168), mRNA	XM_018158661.1	82,4	2,00E-11	87,69%
OG0040020	PREDICTED: Hyalella azteca uncharacterized LOC108666458 (LOC108666458), mRNA	XM_018153338.1	576	4,00E-160	84,39%
OG0040037	PREDICTED: Hyalella azteca integrator complex subunit 3-like (LOC108676217), mRNA	XM_018164271.1	756	0	92,32%
OG0040063	PREDICTED: Hyalella azteca uncharacterized LOC108672394 (LOC108672394), ncRNA	XR_001930890.1	410	4,00E-110	77,59%
OG0040137	PREDICTED: Hyalella azteca kinesin-like protein KIF14 (LOC108676295), mRNA	XM_018164355.1	241	4,00E-59	83,33%
OG0040148	PREDICTED: Hyalella azteca uncharacterized LOC108680352 (LOC108680352), transcript variant X2, mRNA	XM_018169156.1	473	4,00E-129	76,42%
OG0040263	PREDICTED: Hyalella azteca probable G-protein coupled receptor CG31760 (LOC108676810), mRNA	XM_018164948.1	323	1,00E-83	78,00%
OG0040271	PREDICTED: Hyalella azteca fork head domain-containing protein FD5-like (LOC108677215), mRNA	XM_018165404.1	705	0	91,00%
OG0040273	-	-	-	-	-
OG0040293	PREDICTED: Hyalella azteca haloacid dehalogenase-like hydrolase domain-containing protein 3 (LOC108682048), mRNA	XM_018171152.1	685	0	85,00%
OG0040304	-	-	-	-	-
OG0040309	-	-	-	-	-
OG0040312	-	-	-	-	-
OG0040350	PREDICTED: Hyalella azteca TWiK family of potassium channels protein 12-like (LOC108677541), mRNA	XM_018165770.1	469	1,00E-127	86,00%
OG0040405	PREDICTED: Hyalella azteca dynein heavy chain 5, axonemal-like (LOC108673426), mRNA	XM_018161252.1	316	4,00E-82	87,00%
OG0040445	PREDICTED: Hyalella azteca neurogenic locus Notch protein-like (LOC108682494), mRNA	XM_018171660.1	675	0	89,00%
OG0040473	PREDICTED: Hyalella azteca uncharacterized LOC108670316 (LOC108670316), mRNA	XM_018157778.1	452	2,00E-112	75,00%
OG0040490	PREDICTED: Hyalella azteca transducin-like enhancer protein 4 (LOC108678998), transcript variant X5, mRNA	XM_018167501.1	502	4,00E-138	95,00%
OG0040491	PREDICTED: Hyalella azteca transducin-like enhancer protein 4 (LOC108678998), transcript variant X5, mRNA	XM_018167501.1	629	6,00E-176	94,00%
OG0040615	PREDICTED: Hyalella azteca uncharacterized LOC108681384 (LOC108681384), mRNA	XM_018170403.1	663	0	87,00%
OG0040641	-	-	-	-	-
OG0040650	-	-	-	-	-
OG0040678	PREDICTED: Hyalella azteca uncharacterized LOC108674626 (LOC108674626), mRNA	XM_018162589.1	223	1,00E-53	73,00%
OG0040696	PREDICTED: Hyalella azteca serine-rich adhesin for platelets-like (LOC108668707), mRNA	XM_018155952.1	289	6,00E-74	90,00%
OG0040765	PREDICTED: Hyalella azteca uncharacterized LOC108668354 (LOC108668354), transcript variant X1, mRNA	XM_018155550.1	710	0	92,00%
OG0040774	PREDICTED: Hyalella azteca fibrillin-2-like (LOC108669757), mRNA	XM_018157161.1	727	0	87,00%
OG0040780	PREDICTED: Hyalella azteca dmX-like protein 2 (LOC108673863), transcript variant X2, mRNA	XM_018161742.1	682	0	88,00%
OG0040790	PREDICTED: Hyalella azteca uncharacterized LOC108679752 (LOC108679752), mRNA	XM_018168470.1	519	8,00E-143	88,00%
OG0040793	PREDICTED: Hyalella azteca uncharacterized LOC108673275 (LOC108673275), mRNA	XM_018161076.1	269	2,00E-67	81,00%
OG0040806	PREDICTED: Hyalella azteca proclotting enzyme-like (LOC108674941), transcript variant X2, mRNA	XM_018162930.1	315	2,00E-81	76,00%
OG0040867	PREDICTED: Hyalella azteca uncharacterized LOC108671795 (LOC108671795), mRNA	XM_018159378.1	438	3,00E-118	86,00%
OG0040966	PREDICTED: Hyalella azteca clustered mitochondria protein homolog (LOC108665963), transcript variant X2, mRNA	XM_018152772.1	474	3,00E-129	85,00%
OG0040969	PREDICTED: Hyalella azteca uncharacterized LOC108675850 (LOC108675850), transcript variant X4, mRNA	XM_018163893.1	627	2,00E-175	89,00%
OG0040979	PREDICTED: Hyalella azteca uncharacterized LOC108664918 (LOC108664918), mRNA	XM_018151615.1	317	5,00E-82	77,00%
OG0040983	PREDICTED: Hyalella azteca cAMP-specific 3',5'-cyclic phosphodiesterase 4C-like (LOC108668480), transcript variant X5, mRNA	XM_018155711.1	554	1,00E-153	86,00%
OG0040996	-	-	-	-	-
OG0041015	PREDICTED: Hyalella azteca uncharacterized LOC108677619 (LOC108677619), mRNA	XM_018165874.1	394	5,00E-94	78,00%
OG0041023	PREDICTED: Hyalella azteca H2.0-like homeobox protein (LOC108674925), transcript variant X2, mRNA	XM_018162908.1	428	1,00E-115	80,00%
OG0041028	PREDICTED: Hyalella azteca uncharacterized LOC108673243 (LOC108673243), mRNA	XM_018161042.1	448	1,00E-121	80,00%
OG0041069	PREDICTED: Hyalella azteca FMRFamide receptor-like (LOC108681239), partial mRNA	XM_018170252.1	445	1,00E-120	88,00%
OG0041070	PREDICTED: Hyalella azteca FMRFamide receptor-like (LOC108681239), partial mRNA	XM_018170252.1	485	1,00E-132	89,00%
OG0041071	PREDICTED: Hyalella azteca delta-1-pyrroline-5-carboxylate synthase-like (LOC108668397), mRNA	XM_018155603.1	446	3,00E-121	87,00%
OG0041072	-	-	-	-	-
OG0041081	-	-	-	-	-
OG0041109	-	-	-	-	-
OG0041182	PREDICTED: Hyalella azteca MAP7 domain-containing protein 1-like (LOC108680118), transcript variant X9, mRNA	XM_018168883.1	370	2,00E-98	82,00%
OG0041191	PREDICTED: Hyalella azteca uncharacterized LOC108665291 (LOC108665291), transcript variant X3, mRNA	XM_018152027.1	719	0	84,00%
OG0041201	-	-	-	-	-
OG0041218	PREDICTED: Hyalella azteca uncharacterized LOC108680896 (LOC108680896), transcript variant X2, mRNA	XM_018169823.1	305	4,00E-78	73,00%
OG0041223	-	-	-	-	-
OG0041234	PREDICTED: Hyalella azteca merlin-like (LOC108682175), mRNA	XM_018171298.1	420	5,00E-113	91,00%
OG0041259	-	-	-	-	-
OG0041260	-	-	-	-	-
OG0041368	PREDICTED: Hyalella azteca uncharacterized LOC108682652 (LOC108682652), mRNA	XM_018171857.1	439	4,00E-119	91,00%
OG0041369	PREDICTED: Hyalella azteca uncharacterized LOC108674500 (LOC108674500), partial mRNA	XM_018162458.1	587	2,00E-163	83,00%
OG0041384	PREDICTED: Thrips palmi teneurin-a-like (LOC117643064), mRNA	XM_034381714.1	136	5,00E-28	77,00%
OG0041385	PREDICTED: Hyalella azteca B-cell CLL/lymphoma 6 member B protein-like (LOC108676330), mRNA	XM_018164394.1	619	3,00E-173	91,00%
OG0041395	PREDICTED: Hyalella azteca sodium-dependent proline transporter-like (LOC108666718), mRNA	XM_018153634.1	439	2,00E-118	81,00%
OG0041435	PREDICTED: Hyalella azteca uncharacterized LOC108680218 (LOC108680218), mRNA	XM_018169002.1	434	4,00E-117	77,00%
OG0041437	-	-	-	-	-
OG0041446	PREDICTED: Hyalella azteca serine/threonine-protein kinase pakG-like (LOC108681676), mRNA	XM_018170727.1	353	6,00E-93	90,00%
OG0041482	-	-	-	-	-
OG0041509	PREDICTED: Hyalella azteca myb-like protein AA (LOC108667809), mRNA	XM_018154879.1	486	4,00E-133	84,00%
OG0041558	PREDICTED: Hyalella azteca gonadotropin-releasing hormone II receptor-like (LOC108667458), mRNA	XM_018154483.1	671	0	85,00%
OG0041592	PREDICTED: Hyalella azteca uncharacterized LOC108667130 (LOC108667130), mRNA	XM_018154116.1	638	1,00E-178	87,00%
OG0041661	PREDICTED: Hyalella azteca uncharacterized LOC108680144 (LOC108680144), mRNA	XM_018168916.1	165	2,00E-36	83,00%
OG0041664	PREDICTED: Hyalella azteca CLK4-associating serine/arginine rich protein-like (LOC108672277), transcript variant X3, mRNA	XM_018159918.1	563	9,00E-156	84,00%
OG0041672	PREDICTED: Hyalella azteca condensin complex subunit 3-like (LOC108679158), mRNA	XM_018167742.1	276	1,00E-69	87,00%
OG0041743	PREDICTED: Hyalella azteca uncharacterized LOC108671460 (LOC108671460), mRNA	XM_018159013.1	373	7,00E-99	80,00%
OG0041759	-	-	-	-	-
OG0041790	PREDICTED: Hyalella azteca allatostatin-A receptor-like (LOC108668734), mRNA	XM_018155976.1	554	4,00E-153	89,00%
OG0041876	PREDICTED: Hyalella azteca fat-like cadherin-related tumor suppressor homolog (LOC108683064), partial mRNA	XM_018172343.1	288	2,00E-73	86,00%
OG0041964	-	-	-	-	-

Gene Code	missing species	n_species	Mean Lenght (bp)	After Trimming (bp)	constant	parsimony	distinct_si	freqA	freqC	freqG	freqT	Missing data
OG0039918	missing CHL_1	36	405	222	119	73	118	0,3284	0,1917	0,2322	0,2477	3,40%
OG0039932	none	37	361	366	258	71	143	0,3132	0,1993	0,2434	0,2441	6,20%
OG0040004	none	37	586	621	453	93	220	0,2434	0,2287	0,2609	0,2669	8,20%
OG0040013	none	37	434	414	262	100	180	0,2702	0,2175	0,2077	0,3046	8,10%
OG0040020	none	37	499	540	400	92	215	0,2729	0,2682	0,2287	0,2302	11,70%
OG0040037	none	37	499	528	429	64	157	0,3731	0,2011	0,2253	0,2004	9,50%
OG0040063	missing azteca	36	555	534	360	128	192	0,2979	0,2196	0,1948	0,2877	5,90%
OG0040137	none	37	542	498	302	142	218	0,27	0,2283	0,252	0,2497	2,60%
OG0040148	missing azteca	36	521	636	404	145	270	0,2582	0,2177	0,217	0,3071	18,80%
OG0040263	none	37	493	465	246	155	263	0,2894	0,2384	0,2317	0,2405	11,40%
OG0040271	none	37	452	438	341	60	141	0,2049	0,3485	0,1542	0,2924	8,10%
OG0040273	none	37	1044	1116	685	278	454	0,391	0,2065	0,2332	0,1693	14,60%
OG0040293	none	37	561	600	404	135	244	0,259	0,22	0,2271	0,2939	11,00%
OG0040304	missing azteca	36	422	462	310	124	222	0,2503	0,254	0,227	0,2686	12,30%
OG0040309	missing azteca	36	376	276	232	37	76	0,2262	0,238	0,2473	0,2886	4,30%
OG0040312	none	37	384	372	242	93	179	0,215	0,2652	0,2165	0,3033	9,50%
OG0040350	missing AP18	36	456	438	313	74	163	0,2212	0,3125	0,2171	0,2492	7,40%
OG0040405	none	37	385	330	244	61	124	0,2434	0,2456	0,2494	0,2615	8,50%
OG0040445	none	37	444	417	318	57	155	0,2291	0,2814	0,2546	0,2348	11,40%
OG0040473	none	37	540	549	283	175	316	0,3118	0,223	0,2555	0,2098	12,60%
OG0040490	missing 3TK16A	36	430	318	280	29	60	0,2334	0,2633	0,2601	0,2432	6,10%
OG0040491	missing azteca	36	397	408	364	39	69	0,2336	0,2686	0,259	0,2387	12,80%
OG0040615	none	37	542	501	369	90	193	0,2586	0,304	0,2503	0,1871	13,40%
OG0040641	none	37	472	474	380	52	146	0,2333	0,2908	0,2548	0,2211	7,80%
OG0040650	Missing 4816	36	535	552	316	161	252	0,3118	0,1954	0,23	0,2628	6,20%
OG0040678	missing azteca	36	533	546	312	183	266	0,2484	0,295	0,2398	0,2167	11,70%
OG0040696	none	37	392	228	172	36	75	0,2659	0,2656	0,264	0,2045	1,80%
OG0040765	missing azteca	36	462	462	391	57	144	0,2479	0,2592	0,2196	0,2733	13,20%
OG0040774	none	37	548	606	479	85	180	0,214	0,2708	0,2864	0,2288	16,00%
OG0040780	missing azteca	36	475	498	373	58	156	0,2187	0,2856	0,2217	0,274	11,40%
OG0040790	none	37	446	420	263	108	185	0,2896	0,2128	0,2266	0,271	5,40%
OG0040793	none	37	426	456	267	113	243	0,1942	0,2943	0,2165	0,295	12,90%
OG0040806	none	37	446	462	257	139	243	0,3139	0,257	0,2032	0,226	9,70%
OG0040867	none	37	534	510	293	112	266	0,3371	0,2369	0,2183	0,2077	11,40%
OG0040966	missing azteca	36	394	408	297	89	155	0,2617	0,2497	0,18	0,3087	8,00%
OG0040969	none	37	563	648	491	117	236	0,2605	0,2867	0,2451	0,2077	16,50%
OG0040979	none	37	566	612	351	167	316	0,3055	0,2194	0,2425	0,2326	13,50%
OG0040983	none	37	431	456	326	70	179	0,2794	0,1816	0,2496	0,2894	8,00%
OG0040996	missing azteca	36	418	447	295	119	211	0,228	0,2235	0,1983	0,3502	11,50%
OG0041015	none	37	561	570	349	125	270	0,2489	0,3054	0,1764	0,2693	7,50%
OG0041023	none	37	410	459	277	103	237	0,3218	0,2892	0,1649	0,224	15,50%
OG0041028	none	37	508	528	316	128	258	0,2576	0,2739	0,2406	0,2278	11,20%
OG0041069	missing azteca	36	358	315	253	50	88	0,1584	0,2929	0,294	0,2548	3,30%
OG0041070	none	37	450	366	281	62	114	0,1769	0,2855	0,2853	0,2523	4,10%
OG0041071	none	37	388	381	267	89	161	0,2474	0,2196	0,2249	0,308	9,00%
OG0041072	missing azteca	36	420	432	362	47	109	0,2419	0,2754	0,2308	0,2519	5,70%
OG0041081	none	37	307	294	222	51	85	0,293	0,198	0,268	0,241	1,80%
OG0041109	missing azteca	36	328	330	214	90	133	0,1861	0,2369	0,2365	0,3405	4,30%
OG0041182	missing azteca	36	314	387	280	72	153	0,2217	0,2842	0,2031	0,291	21,80%
OG0041191	missing Ecu_loc02	36	610	543	374	97	215	0,2118	0,233	0,2236	0,3315	6,70%
OG0041201	none	37	399	393	164	150	234	0,2709	0,2444	0,2276	0,2571	4,00%
OG0041218	none	37	462	519	212	187	346	0,2695	0,2328	0,2108	0,2869	16,00%
OG0041223	none	37	575	618	452	102	211	0,2154	0,2955	0,2321	0,257	11,00%
OG0041234	none	37	403	306	237	50	80	0,3289	0,2479	0,2182	0,2051	3,60%
OG0041259	missing azteca	36	469	384	262	100	156	0,2063	0,2732	0,3195	0,201	12,20%
OG0041260	none	37	675	528	310	146	262	0,3072	0,2356	0,2179	0,2393	8,80%
OG0041368	none	37	412	381	313	48	108	0,2577	0,2309	0,2331	0,2782	9,70%
OG0041369	missing azteca	36	523	561	449	86	151	0,2553	0,2197	0,2168	0,3083	7,40%
OG0041384	missing azteca	36	327	243	167	47	85	0,1724	0,3657	0,2638	0,1981	2,80%
OG0041385	none	37	404	354	280	42	97	0,1905	0,2427	0,2491	0,3177	5,60%
OG0041395	none	37	473	450	276	115	204	0,2749	0,2545	0,2525	0,2181	4,50%
OG0041435	missing azteca	36	609	582	319	177	295	0,3218	0,2243	0,2249	0,229	7,30%
OG0041437	none	37	566	558	292	159	285	0,2887	0,2324	0,2279	0,251	6,70%
OG0041446	missing 26_2A, CHL_1, azteca	34	373	300	240	48	93	0,2359	0,295	0,234	0,2352	4,60%
OG0041482	none	37	402	423	251	73	184	0,2749	0,2477	0,2448	0,2326	8,50%
OG0041509	missing 2310E	36	491	480	301	112	229	0,1697	0,2399	0,219	0,3714	10,60%
OG0041558	none	37	566	600	430	109	236	0,2259	0,2477	0,2399	0,2866	14,00%
OG0041592	missing azteca	36	469	456	382	56	119	0,2925	0,2825	0,2404	0,1847	7,70%
OG0041661	none	37	406	282	160	70	140	0,2311	0,2875	0,2272	0,2541	5,90%
OG0041664	none	37	477	516	360	63	201	0,2918	0,2839	0,2856	0,1387	15,60%
OG0041672	none	37	373	270	186	62	99	0,2082	0,2705	0,2495	0,2718	3,30%
OG0041743	none	37	455	495	312	121	249	0,2366	0,2888	0,2628	0,2117	13,80%
OG0041759	none	37	419	396	285	52	132	0,2665	0,2376	0,2096	0,2863	9,60%
OG0041790	none	37	510	510	377	81	176	0,2485	0,2858	0,2315	0,2343	9,30%
OG0041876	missing azteca	36	374	261	204	43	76	0,1996	0,3196	0,2834	0,1974	3,30%
OG0041964	Missing 4816, azteca	35	479	252	181	32	101	0,2586	0,2895	0,224	0,2279	11,20%

Supplementary Table 3. Single-copy nuclear gene sequences found with Orthofinder. The first column corresponds to the code assigned by the program. Details of the sequences found and corresponding aligments are shown. The "-" symbol, indicates that no significant match was found in the database.

ortholog code	logL	deltaL	p-SH
OG0039918	-1100,17	35,559	0,0165
OG0039932	-1383,15	41,206	0,043
OG0040004	-2263,12	75,167	0,004
OG0040013	-1712,69	55,587	0,00195
OG0040020	-2022,87	67,631	0,00765
OG0040037	-1525,17	36,419	0,0347
OG0040063	-2384,78	100,37	0,00005
OG0040137	-2381,97	76,039	0,002
OG0040148	-2339,76	36,907	0,0221
OG0040263	-2550,94	42,98	0,016
OG0040271	-1456,55	65,089	0,0161
OG0040273	-4803,37	111,63	0,0002
OG0040293	-2360,47	30,216	0,0335
OG0040304	-2016,38	136,94	0,0004
OG0040309	-726,997	18,933	0,0143
OG0040312	-1606,72	59,16	0,0111
OG0040350	-1651,62	45,513	0,00905
OG0040405	-1173,77	32,236	0,0121
**OG0040445**	**-1329,39**	**15,471**	**0,0852**
OG0040473	-3091,04	67,849	0,0173
OG0040490	-819,797	43,181	0,00465
OG0040491	-1041,81	40,698	0,0017
OG0040615	-1890,1	52,946	0,0035
OG0040641	-1429,52	34,497	0,0133
OG0040650	-2556,97	42,9	0,00235
OG0040678	-2720,11	55,066	0,00135
OG0040696	-826,473	37,221	0,0168
OG0040765	-1298,61	50,817	0,007
OG0040774	-1840,19	24,254	0,0158
OG0040780	-1608,85	86,013	0,00035
OG0040790	-2078,47	76,901	0,00295
OG0040793	-1988,76	87,602	0,00225
OG0040806	-2298,39	94,96	0,00125
OG0040867	-2261,26	39,644	0,033
OG0040966	-1382,42	25,321	0,0282
OG0040969	-2253,12	78,099	0,0061
OG0040979	-3083,09	90,865	0,00085
**OG0040983**	**-1594,15**	**30,607**	**0,0566**
OG0040996	-2002,86	119,38	0,00105
OG0041015	-2425,63	38,822	0,0112
OG0041023	-1967,4	35,212	0,0347
OG0041028	-2310,47	26,999	0,0341
OG0041069	-1020,35	38,571	0,00995
OG0041070	-1317,73	50,031	0,0035
OG0041071	-1620,54	139,42	0
OG0041072	-1196,33	27,197	0,0159
OG0041081	-1059,52	56,172	0,00715
OG0041109	-1623,28	99,669	0,00005
OG0041182	-1350,64	96,73	0,00325
OG0041191	-2281,49	61,522	0,0044
OG0041201	-2430,61	73,512	0,0091
OG0041218	-3142,23	55,046	0,0147
OG0041223	-2151,19	70,492	0,019
OG0041234	-1005,35	41,477	0,0241
**OG0041259**	**-1451,88**	**10,207**	**0,0766**
OG0041260	-2629,53	112,81	0,00365
**OG0041368**	**-1109,11**	**10,598**	**0,0707**
OG0041369	-1633,89	25,57	0,0362
OG0041384	-1147,11	100,95	0
OG0041385	-1030,11	22,143	0,0273
**OG0041395**	**-1969,05**	**36,287**	**0,0616**
OG0041435	-3064,05	128,93	0,00115
OG0041437	-2953,48	78,245	0,013
OG0041446	-873,395	30,819	0,0395
OG0041482	-1806,27	24,409	0,0339
OG0041509	-2079,74	52,767	0,0168
OG0041558	-2269,27	60,358	0,0132
**OG0041592**	**-1209,33**	**22,376**	**0,0919**
OG0041661	-1418,04	16,419	0,0486
OG0041664	-1753	47,134	0,00505
OG0041672	-1117,89	68,22	0,00455
OG0041743	-2161,15	29,862	0,0326
**OG0041759**	**-1288,4**	**12,098**	**0,0603**
OG0041790	-1832,84	61,452	0,00905
OG0041876	-916,77	47,487	0,00465
OG0041964	-849,877	30,735	0,0449

Supplementary Table 4. Shimodaira-Hasegawa tests contrasting ML topologies obtained with each single-copy nuclear gene-fragment versus the concatenated ML tree. p-SH = significance of the Shimodaira-Hasegawa test. Cells in blue indicate P > 0.05 for p-SH.
